# Developmental organization of neural dynamics supporting auditory perception

**DOI:** 10.1016/j.neuroimage.2022.119342

**Published:** 2022-05-30

**Authors:** Kazuki Sakakura, Masaki Sonoda, Takumi Mitsuhashi, Naoto Kuroda, Ethan Firestone, Nolan O’Hara, Hirotaka Iwaki, Min-Hee Lee, Jeong-Won Jeong, Robert Rothermel, Aimee F. Luat, Eishi Asano

**Affiliations:** a Department of Pediatrics, Children’s Hospital of Michigan, Detroit Medical Center, Wayne State University, Detroit, MI, 48201, USA; b Department of Neurology, Children’s Hospital of Michigan, Detroit Medical Center, Wayne State University, Detroit, Michigan, 48201, USA; c Translational Neuroscience Program, Wayne State University, Detroit, Michigan, 48201, USA; d Department of Physiology, Wayne State University, Detroit, MI 48201, USA; e Department of Psychiatry, Children’s Hospital of Michigan, Detroit Medical Center, Wayne State University, Detroit, Michigan, 48201, USA; f Department of Neurosurgery, University of Tsukuba, Tsukuba, 3058575, Japan; g Department of Neurosurgery, Yokohama City University, Yokohama, Kanagawa, 2360004, Japan; h Department of Neurosurgery, Juntendo University, School of Medicine, Tokyo, 1138421, Japan; i Department of Epileptology, Tohoku University Graduate School of Medicine, Sendai 9808575, Japan; j Department of Pediatrics, Central Michigan University, Mt. Pleasant, MI 48858, USA

**Keywords:** Ontogeny, Language acquisition, Intracranial electroencephalography (EEG) recording, Event-related high-gamma synchronization, Physiological high-frequency oscillations (HFOs), Pediatric epilepsy surgery, Subdural grid electrodes, Electrocorticography (ECoG), Neural pruning, Neurolinguistics

## Abstract

**Purpose:**

A prominent view of language acquisition involves learning to ignore irrelevant auditory signals through functional reorganization, enabling more efficient processing of relevant information. Yet, few studies have characterized the neural spatiotemporal dynamics supporting rapid detection and subsequent disregard of irrelevant auditory information, in the developing brain. To address this unknown, the present study modeled the developmental acquisition of cost-efficient neural dynamics for auditory processing, using intracranial electrocorticographic responses measured in individuals receiving standard-of-care treatment for drug-resistant, focal epilepsy. We also provided evidence demonstrating the maturation of an anterior-to-posterior functional division within the superior-temporal gyrus (STG), which is known to exist in the adult STG.

**Methods:**

We studied 32 patients undergoing extraoperative electrocorticography (age range: eight months to 28 years) and analyzed 2,039 intracranial electrode sites outside the seizure onset zone, interictal spike-generating areas, and MRI lesions. Patients were given forward (normal) speech sounds, backward-played speech sounds, and signal-correlated noises during a task-free condition. We then quantified sound processing-related neural costs at given time windows using high-gamma amplitude at 70–110 Hz and animated the group-level high-gamma dynamics on a spatially normalized three-dimensional brain surface. Finally, we determined if age independently contributed to high-gamma dynamics across brain regions and time windows.

**Results:**

Group-level analysis of noise-related neural costs in the STG revealed developmental enhancement of early high-gamma augmentation and diminution of delayed augmentation. Analysis of speech-related high-gamma activity demonstrated an anterior-to-posterior functional parcellation in the STG. The left anterior STG showed sustained augmentation throughout stimulus presentation, whereas the left posterior STG showed transient augmentation after stimulus onset. We found a double dissociation between the locations and developmental changes in speech sound-related high-gamma dynamics. Early left anterior STG high-gamma augmentation (i.e., within 200 ms post-stimulus onset) showed developmental enhancement, whereas delayed left posterior STG high-gamma augmentation declined with development.

**Conclusions:**

Our observations support the model that, with age, the human STG refines neural dynamics to rapidly detect and subsequently disregard uninformative acoustic noises. Our study also supports the notion that the anterior-to-posterior functional division within the left STG is gradually strengthened for efficient speech sound perception *after birth.*

## 1. Introduction

A prominent view of language acquisition involves learning to ignore irrelevant auditory signals through functional reorganization, enabling more efficient processing of relevant information (Bishop, 1999; 2000). Speech sounds are the most critical auditory stimuli for verbal communication, and infants and toddlers are believed to hear an average of 1461 spoken words per hour (Hart and Risley, 1992). A behavioral study of healthy adults suggests that speech sounds are more readily detectable than unintelligible noises (Brungart et al., 2006). Functional magnetic resonance imaging (fMRI) studies of healthy individuals ranging from infants to adults consistently report that non-speech sounds, compared to speech sounds of the same intensity, elicit less intense and less extensive hemodynamic activations in the lateral superior-temporal gyrus (STG) of each hemisphere (Vouloumanos et al., 2001; Desai et al., 2005; Tremblay et al., 2013; Shultz et al., 2014). Reduction of neuronal responses to non-speech sounds may be attributed to the developmental reduction of irrelevant synaptic connections (i.e., a phenomenon known as ‘neural pruning’; Huttenlocher and Dabholkar, 1997; Jernigan et al., 2016). Based on cross-sectional studies using postmortem examination and positron emission tomography from those with focal epilepsy, investigators have suggested that the human auditory cortex’s synaptic density and cortical glucose metabolism peak during infancy and toddlerhood and are then reduced through childhood and adulthood (Chugani et al., 1987; Huttenlocher and Dabholkar, 1997). These behavioral, postmortem, and functional imaging studies provide evidence supporting the aforementioned view of language acquisition proposed by Bishop (Bishop, 1999; 2000). However, few studies have characterized the spatiotemporal dynamics supporting real-time *rapid* detection and *subsequent* disregard of irrelevant auditory information in the developing brain. Our present study modeled the developmental acquisition of cost-efficient neural dynamics for auditory perceptual processing by addressing the following two aims.

**Fig. 1.**
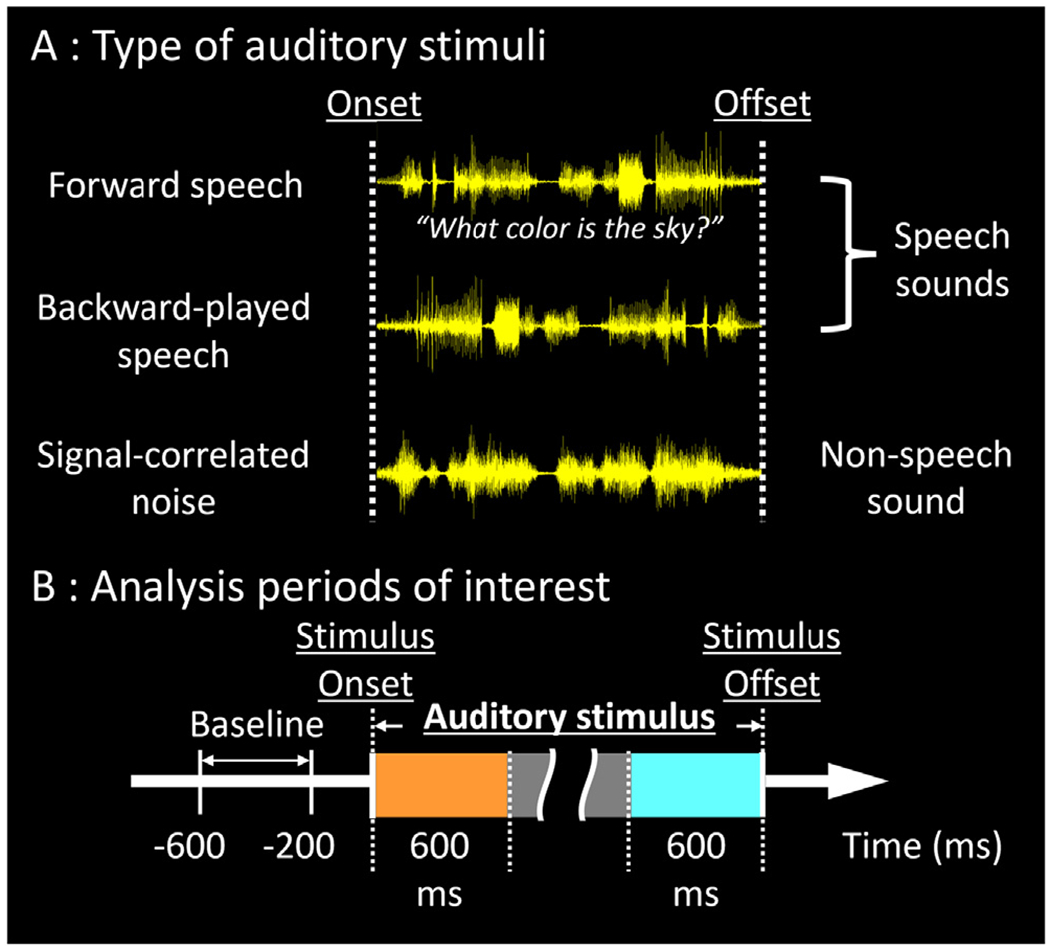
Sound stimuli and analysis time windows. (A) forward speech, (B) backward-played speech, and (C) signal-correlated noise sound waves. The analysis periods of interest included (D) a 600-ms period immediately after the stimulus onset (colored in orange) and (E) another 600-ms immediately before the stimulus offset (colored in light blue).

**[Aim 1]** The first aim characterized the ontogenic changes of neural dynamics associated with auditory processing of unintelligible noises. We hypothesized that the *lateral STG* would acquire and strengthen the ability to *rapidly* detect/analyze auditory information and *subsequently* discard information unrelated to spoken language, to reduce the overall neural costs for auditory perception. This hypothesis was in part motivated by non-invasive neuroimaging and electrophysiology observations. Several fMRI studies reported that speech sounds, compared to non-speech sounds, elicited greater hemodynamic responses in the STG (Vouloumanos et al., 2001; Tremblay et al., 2013). A longitudinal fMRI study reported that left STG hemodynamic responses induced by acoustic sine-wave stimuli were enhanced after training in individuals who began perceiving these synthetic stimuli as speech sounds, compared to those who kept perceiving them as non-speech sounds (Möttönen et al., 2006). These fMRI observations suggest that speech sounds may be associated with higher neural costs in the STG. Thus, we expected the present study to demonstrate that the STG neural cost for processing unintelligible noises (Fig. 1) would be smaller than for speech sounds (Fig. 1). Scalp electroencephalography (EEG) studies of healthy individuals, ranging from infants to young adults, infer the timing during which the human cerebral cortex distinguishes between speech and non-speech sounds. Auditory event-related potential (ERP) waveform deflections elicited by repeated and deviant phoneme or non-speech stimuli were reported to be comparable at 100 ms post-stimulus onset but became distinct at 150–250 ms and later (Kraus and McGee, 1994; Maurer et al., 2003; Kozou et al., 2005; Sorokin et al., 2010; Paquette et al., 2013; Christmann et al., 2014; Ortiz-Mantilla et al., 2016; Galilee et al., 2017). We tested the hypothesis that noise-related STG neural cost at an *early* period (i.e., within 200 ms post-stimulus onset) would be enhanced in an age-dependent manner, but it would be diminished at a *delayed* period (i.e., >200 ms post-stimulus onset).

To address **[Aim 1]**, we quantified the neural cost at given areas and time windows using event-related high-gamma activity at 70–110 Hz recorded on intracranial EEG (iEEG). The degree of high-gamma augmentation is an outstanding summary measure of event-related neural cost. Enhanced high-gamma amplitude is tightly associated with an increased firing rate on single-neuron recording (Ray et al., 2008; Manning et al., 2009), hemodynamic activation on fMRI (Scheeringa et al., 2011; Hermes et al., 2012), and glucose metabolism on positron emission tomography (Nishida et al., 2008). Damage to cortical sites showing naming-related high-gamma augmentation increase the risk of language deficits following resective surgery (Kojima et al., 2013); furthermore, naming-related augmentation of high-gamma activity, compared to other frequency bands, is more predictive of post-operative language outcomes (Sonoda et al., 2022). On the other hand, diminished high-gamma augmentation is suggested to reflect lower neural costs (Shmuel et al., 2006; Matsuzaki et al., 2012; Engell and McCarthy, 2014; Rodriguez Merzagora et al., 2014; Korzeniewska et al., 2020).

**[Aim 2]** We aimed to provide evidence of the developmental organization of the anterior-to-posterior functional division as reported to exist in the adult STG (Ozker et al., 2017, 2018; Hamilton et al., 2018). Small cohort iEEG studies of adult patients with focal epilepsy reported a sharp, functional boundary in the lateral STG (Hamilton et al., 2018; Ozker et al., 2017, 2018). The *anterior* STG (defined as that anterior to the posterior margin of Heschl’s gyrus) reportedly showed high-gamma activity augmented more by speech sounds, compared to noises (Ozker et al., 2017, 2018). The anterior STG is suggested to encode phonetic features based on its sustained high-gamma augmentation during presentation of spoken sentences, whereas the posterior STG is suggested to decode the boundary between sound series based on the transient high-gamma augmentation immediately after the onset of spoken sentences (Hamilton et al., 2018). The current study aimed to determine whether such an anterior-to-posterior functional division within the STG strengthens with age, which would be consistent with the language acquisition view proposed by Bishop (Bishop, 1999; 2000). We hypothesized that speech sounds would initially be processed equally in both the anterior and posterior STG, but with development, the posterior STG would become more specialized for detecting the onset of spoken sentences between sound series, as previously suggested (Hamilton et al., 2018). We determined whether older individuals would show diminished speech sound-related posterior STG high-gamma augmentation during a delayed post-stimulus period.

## 2. Material and methods

### 2.1. Participants

The inclusion criteria consisted of (i) extraoperative iEEG recording at Children’s Hospital of Michigan or Harper University Hospital, Detroit Medical Center between April 2012 and September 2018, (ii) iEEG signal sampling at least from the lateral temporal lobe, and (iii) measurement of sound-related high-gamma responses under an interictal, task-free condition, as described below. The exclusion criteria included (i) massive brain malformations deforming the central or lateral sulcus (Nakai et al., 2017), (ii) history of previous resective epilepsy surgery (Nakai et al., 2017), (iii) history of hearing deficits, and (iv) left-handedness associated with congenital neocortical lesions in the left hemisphere (because of the high probability of right-hemispheric language dominance; Rasmussen and Milner, 1977; Akanuma et al., 2003; Möddel et al., 2009; Kojima et al., 2013; Sonoda et al., 2022). The Institutional Review Board at Wayne State University approved the present study. We obtained informed consent from the legal guardians of patients and assent from pediatric patients.

### 2.2. Intracranial electrode placement and extraoperative intracranial eeg (iEEG) data acquisition

As a part of the standard-of-care management of drug-resistant focal seizures, we implanted platinum disk macroelectrodes (center-to-center distance: 10 mm; diameter: 3 mm) in the subdural space to characterize the boundary between the seizure onset zone and eloquent areas (Nakai et al., 2017; Kuroda et al., 2021; Sonoda et al., 2022). For the next 3–7 days, patients were transferred to the Epilepsy Monitoring Unit, and antiseizure medications were discontinued to facilitate capturing ictal events for localizing the seizure onset zone (Asano et al., 2009). We continuously recorded extraoperative iEEG signals with a rate of 1000 Hz and a band-pass of 0.016–300 Hz, video, electrooculography signals (using electrodes placed 2.5 cm below and 2.5 cm lateral to the left and right outer canthi; Uematsu et al., 2013), and electromyography signals (using electrodes placed on the left and right deltoid muscles; Uematsu et al., 2013). We analyzed iEEG signals using a common average reference consisting of the average iEEG voltage from all electrode channels, excluding those affected by artifacts (Uematsu et al., 2013), seizure onset zone, interictal spikes (Kural et al., 2020), or MRI lesions (Kuroda et al., 2021).

### 2.3. Imaging data acquisition and analysis

**Fig. 2.**
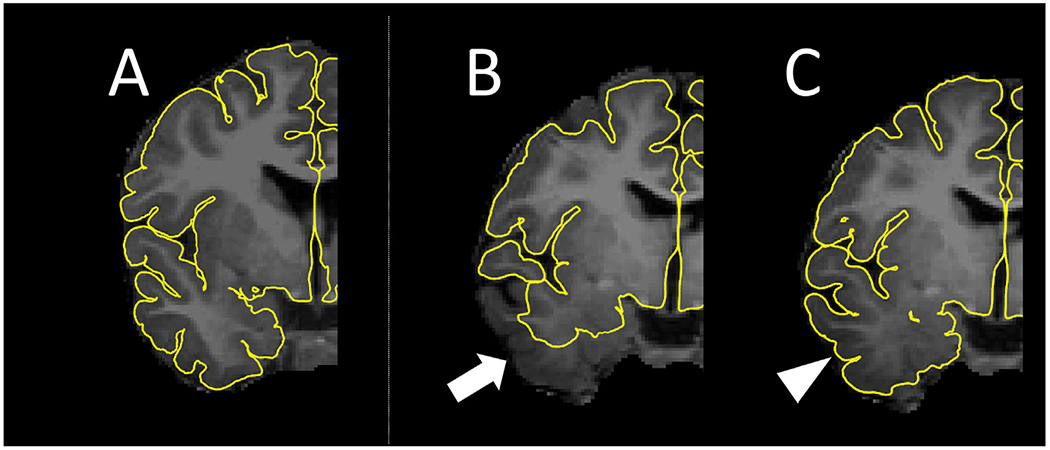
Delineation of the pial surface in young children. (A) The FreeSurfer software package automatically delineated the pial surface in a 3-year-old child, as denoted by yellow lines. (B) The FreeSurfer software package failed to outline the pial surface automatically; the performance was worst in the temporal lobe (arrow) in a 2-year-old child. (C) Using the FreeSurfer Control Point function, therefore, a board-certified neurosurgeon (K.S.) needed to delineate the pial surface manually. This function allowed us to accurately delineate the pial surface, as denoted by an arrowhead.

Before intracranial electrodes were implanted, we acquired 3-tesla MRI scans, including a T1-weighted spoiled gradient-echo volumetric scan and a fluid-attenuated inversion recovery scan (Kuroda et al., 2021). After electrodes were implanted, we acquired a CT scan, as well as lateral and anterior-posterior x-ray images, to visualize intracranial electrode locations. For patients aged two and above, we generated individual three-dimensional brain surface images using the FreeSurfer software package (http://surfer.nmr.mgh.harvard.edu; Ghosh et al., 2010). Due to insufficient cerebrum myelination, especially in the temporal lobe tips, the FreeSurfer software package failed to delineate the pial surface automatically in three children (age ranging from two to three years). In these three patients, the pial surface was manually delineated using the Control Point function implemented in the FreeSurfer software package (https://surfer.nmr.mgh.harvard.edu/fswiki/FsTutorial/ControlPoints_freeview/; Deoni et al., 2015; Croteau-Chonka et al., 2016; Remer et al., 2017; Fig. 2). For those younger than two years, we used the Infant FreeSurfer software package (https://surfer.nmr.mgh.harvard.edu/fswiki/infantFS; Zöllei et al., 2020).

**Fig. 3.**
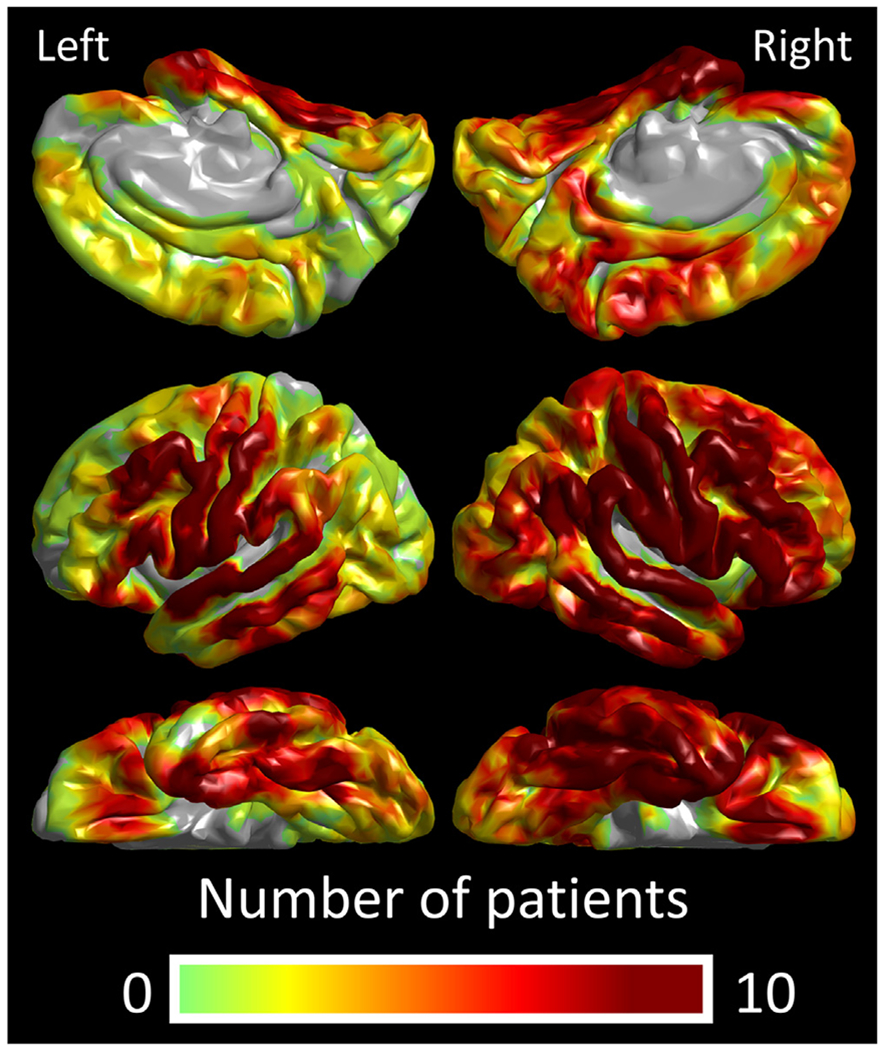
Spatial distribution of intracranial electrode sampling. A total of 2039 non-epileptic subdural electrode sites were available for analysis.

**Fig. 4.**
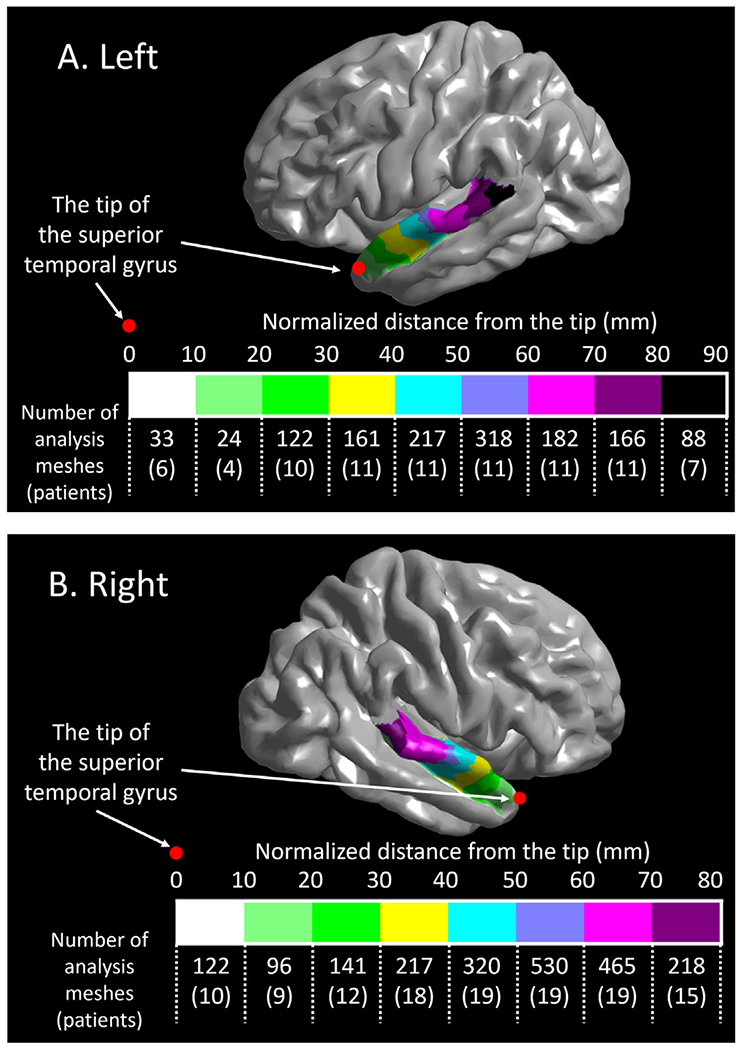
Each region of interest (ROI) in the superior temporal gyrus (STG). (A) The left STG was divided into nine ROIs, as coded in different colors. The most posterior STG electrode site was located at 88.6 mm normalized distance from the temporal lobe tip. (B) The right STG was divided into eight ROIs. The most posterior STG electrode site was located at 79.9 mm normalized distance from the tip. The number of non-epileptic analysis meshes at each ROI is provided (with the number of contributing patients). Each “analysis mesh” consisted of an aggregate of 20 neighboring FreeSurfer vertex finite elements (Desikan et al., 2006). For example, a total of 19 patients contributed iEEG measures to the right STG_50–60_ mm (defined as the right STG 50–60 mm normalized distance from the tip), which had 27.9 analysis meshes per patient on average.

We co-registered intracranial electrodes from all patients with a three-dimensional brain surface image, as previously described (Nakai et al., 2017; Stolk et al., 2018). Two board-certified neurosurgeons (K.S. and N.K.) confirmed the spatial accuracy of electrode locations co-registered to the reconstructed surface image, using intraoperative photographs taken before dural closure and after the reopening (Pieters et al., 2013; Kambara et al., 2018). We spatially normalized all aggregated electrode sites to the FreeSurfer standard brain template, referred to as ‘FSaverage’ (Fig. 3), allowing high-gamma quantification in a common space across patients. With visual assessment, we confirmed that all electrode sites in a given ROI were normalized to the correct, corresponding ROI on the standard brain template. For each electrode site, the FreeSurfer script provided an estimated error in spatial normalization (unit: mm), indicating how accurately a given electrode site in an individual patient’s brain-space was transformed to the standard brain template. We found that the mean error in each patient ranged from 0.30 mm to 0.40 mm. We failed to find a significant correlation between the patient’s age and the mean error in a given patient (Spearman’s rank rho = −0.0016; *p* = 0.99). Regions of interest (ROIs) were defined automatically based on the FreeSurfer Desikan Atlas (Desikan et al., 2006). To characterize the anterior-to-posterior functional division within the STG of each hemisphere, we further divided the STG into sub-compartments by placing a boundary at each 10 mm normalized distance from the temporal lobe tip (Fig. 4). We defined ‘normalized distance’ as the Euclidean distance measured on the ‘FSaverage’ standard brain template.

### 2.4. Presentation of auditory stimuli under a task-free condition

During extraoperative iEEG recording, all patients were presented 30 trials of forward speech sounds, 30 backward-played speech sounds, and 30 signal-correlated noises (Brown et al., 2012, 2014; Fig. 1) under a task-free condition in a quiet room. The sound stimuli were given in a pseudorandom order via an open-field speaker with an intensity of 70 dB and with inter-stimulus intervals ranging from 3 to 5 s. Each forward speech stimulus consisted of a male voice-recorded sentence (sampling rate: 44.1 kHz), whose median duration was 1.8 s (range: 1 to 2.5 s). Each sentence (e.g., *what flies in the sky? when do you eat dinner?)* had natural prosody and commonly began with a *wh*-interrogative (i.e., *what* [verb], *who* [verb], *when do you* [verb], or *where do you* [verb]). Each backward speech stimulus was made from a forward speech and reversed in time using the Cool Edit Pro-version (Syntrillium Software Corp., Phoenix, AZ, USA). The backward speech sound is unintelligible but perceived as a human voice (an example provided in Brown et al., 2014). Each signal-correlated noise was computer-generated with the same duration and frequency spectrum as a given forward speech sound stimulus but with random phases. The signal-correlated noise is unintelligible and does not sound like a human voice (an example provided in Brown et al., 2014).

During sound presentation, the sleep state of given patients was visually determined using simultaneous recordings of video, iEEG, electrooculography, and electromyography (Uematsu et al., 2013; Kuroda et al., 2021). We aimed to control for the independent effect of sleep-state (wakefulness or asleep) on sound-related iEEG high-gamma responses in the statistical analysis described below. The present study assumed that sleep state has measurable effects on high-gamma responses, although studies using single-neuron recording and fMRI reported that sleep had no discernible systematic depressive effect on auditory perceptual processing in the bilateral STG (Issa et al., 2008; Portas et al., 2000).

### 2.5. Quantitative assessment of intracranial eeg (iEEG) high-gamma responses to sound stimuli

**Fig. 5.**
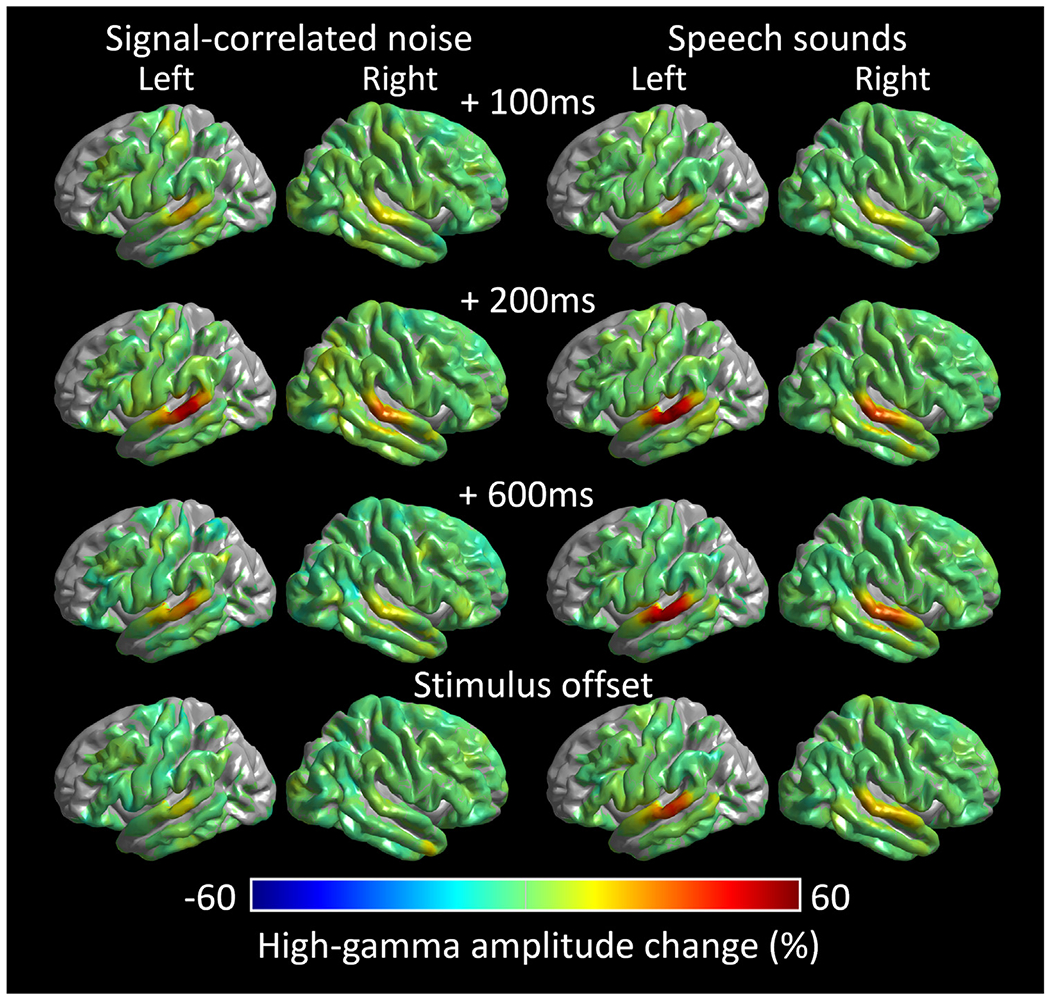
Dynamics of sound-related high-gamma responses. Left: Group-level high-gamma modulations elicited by signal-correlated noises. Right: High-gamma modulations elicited by speech sound stimuli (i.e., average during forward and backward speech sound presentations). (A) 100 ms post-stimulus onset. (B) 200 ms post-stimulus onset. (C) 600 ms post-stimulus onset. (D) stimulus offset. Video S1 shows the high-gamma dynamics at the whole-brain level.

**Fig. 6.**
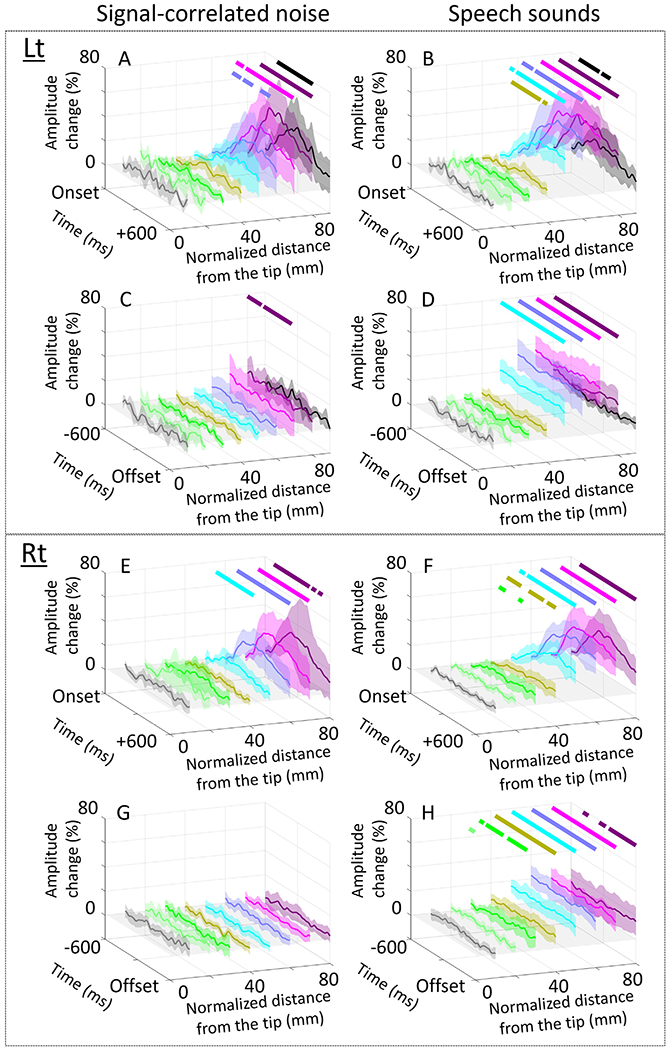
Sound-related high-gamma responses in the superior temporal gyrus (STG). Left: Noise-related high-gamma amplitude (% change) as a function of time (ms) in the left (see A and C) and right STG (E and G). Right: Speech sound-related high-gamma amplitude in the left (B and D) and right STG (F and H). Upper horizontal bars: Significant amplitude augmentation based on the permutation test. Left STG_40–80 mm_ showed *sustained* speech sound-related high-gamma augmentation initiating within 90 ms post-stimulus onset and lasted until the stimulus offset. In contrast, the left STG_80–90 mm_ showed high-gamma augmentation lasting only between 70 and 360 ms post-stimulus onset (see the black horizontal bar in B). Fig. S1 presents the high-gamma dynamics using two-dimensional plots.

The time-frequency analysis (Nakai et al., 2017) was performed at electrode sites both free from non-cerebral artifacts (Sperling, 2003; Kahane and Dubeau, 2014) and deemed to be non-epileptic (defined as those outside the seizure onset zone [Asano et al., 2009], interictal spike-generating areas [Kural et al., 2020], and MRI lesions; Frauscher et al., 2018; Kuroda et al., 2021). At each electrode channel, the iEEG signal was transformed from a time-voltage domain to the time–frequency domain using a complex demodulation program implemented in BESA EEG software (BESA GmbH, Gräfelfing, Germany; Papp and Ktonas, 1977; Hoechstetter et al., 2004; Brown et al., 2012). At each time-frequency bin of 10-ms width and 5-Hz height, we measured the amplitude percent change (proportional to the square root of power) relative to the baseline averaged between 600 and 200 ms before stimulus onset. The time-frequency transformation was done by multiplying the time-domain signal with a complex exponential, followed by a band-pass filter. Since the band-pass filter used here was a finite impulse response filter of Gaussian shape, the complex demodulation was effectively equivalent to a Gabor transform. The filter had a full width at half maximum of 2 × 15.8 ms in the temporal domain and 2 × 7.1 Hz in the frequency domain. Thus, the time-frequency resolution in our analysis was ±15.8 ms and ±7.1 Hz, and defined as the 50% power drop of the finite impulse response filter. The analysis windows included 600 ms immediately after stimulus onset and another 600 ms immediately before stimulus offset (Fig. 1). The percent change in high-gamma (70–110 Hz) amplitude at each electrode channel was interpolated within 10 mm from the electrode center on the FreeSurfer standard brain template (Fig. 5). iEEG signals in this frequency band are unaffected by the effects of alternating current artifacts. To determine the population mean for subsequent statistical analysis, resulting iEEG high-gamma amplitude measures were assigned to all cortical surface mesh points, which each consisted of 20 neighboring FreeSurfer vertex finite elements (Desikan et al., 2006) (Fig. 4, 6, **and** S1).

Statistical analysis of high-gamma amplitudes determined what brain regions were involved in auditory perception. The permutation test (*n* = 1000) evaluated the null hypothesis that the population means of high-gamma responses (% changes in amplitude) to sound stimuli (e.g., speech sounds) would be equal to zero, with a two-sided 5% significance level, followed by a false discovery rate (FDR) correction across the analysis time window alone (i.e., 60 times for 600 ms; Iwaki et al., 2020; Fig. 6 and S1). To address **[Aim 1]**, the permutation test likewise determined at what time windows and ROIs high-gamma responses elicited by signal-correlated noises were smaller than those elicited by speech sounds (i.e., average of those elicited by forward and backward speech sounds), followed by a false discovery rate (FDR) correction across the analysis time window alone (i.e., 60 times for 600 ms). In the present study, we defined significant high-gamma augmentation as the population mean greater than zero during more than three consecutive oscillatory cycles (i.e., FDR-corrected *p*<0.05 for 50 ms or above).

### 2.6. Assessment of the developmental changes in iEEG high-gamma responses

**Fig. 7.**
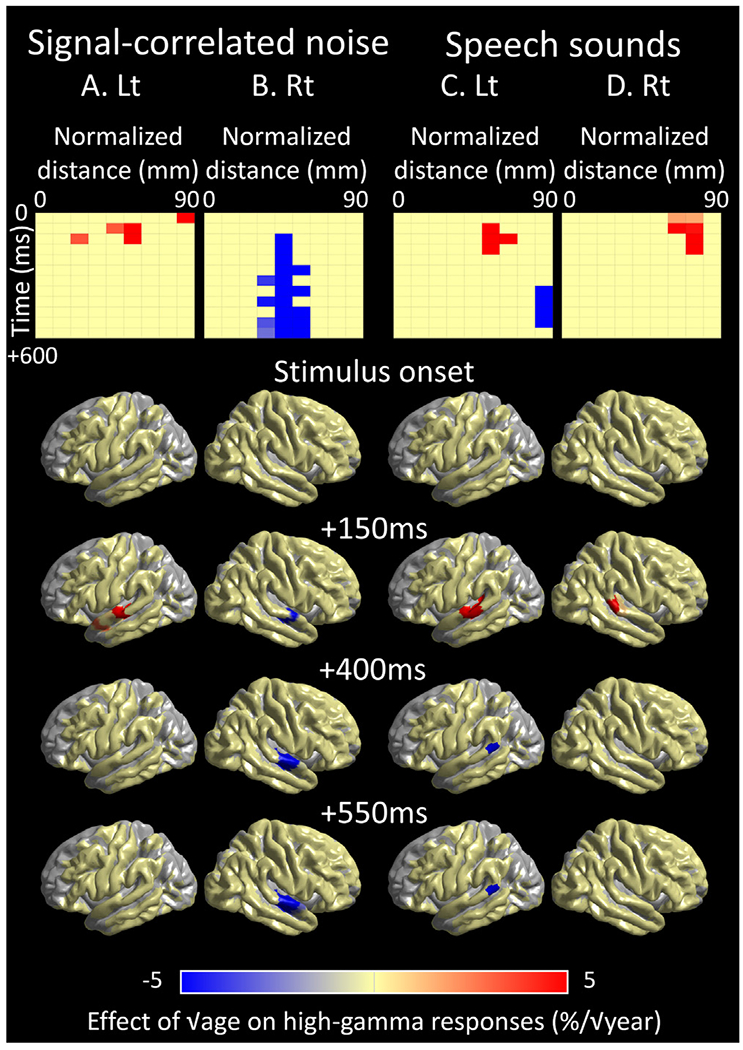
Developmental changes of sound-related high-gamma dynamics in the superior temporal gyrus (STG). Each matrix and brain surface image present the mixed model effect of √age (% / year) on high-gamma amplitude at a given 50-ms time window at each STG region of interest (ROI). (A and B) The significant √age effect on noise-related high-gamma responses in the left and right STG (see the data source in Fig. 8A and 8B). (C and D) The significant √age effect on speech sound-related high-gamma responses in the left and right STG (see the data source in Fig. 8C and 8D).

**Fig. 8.**
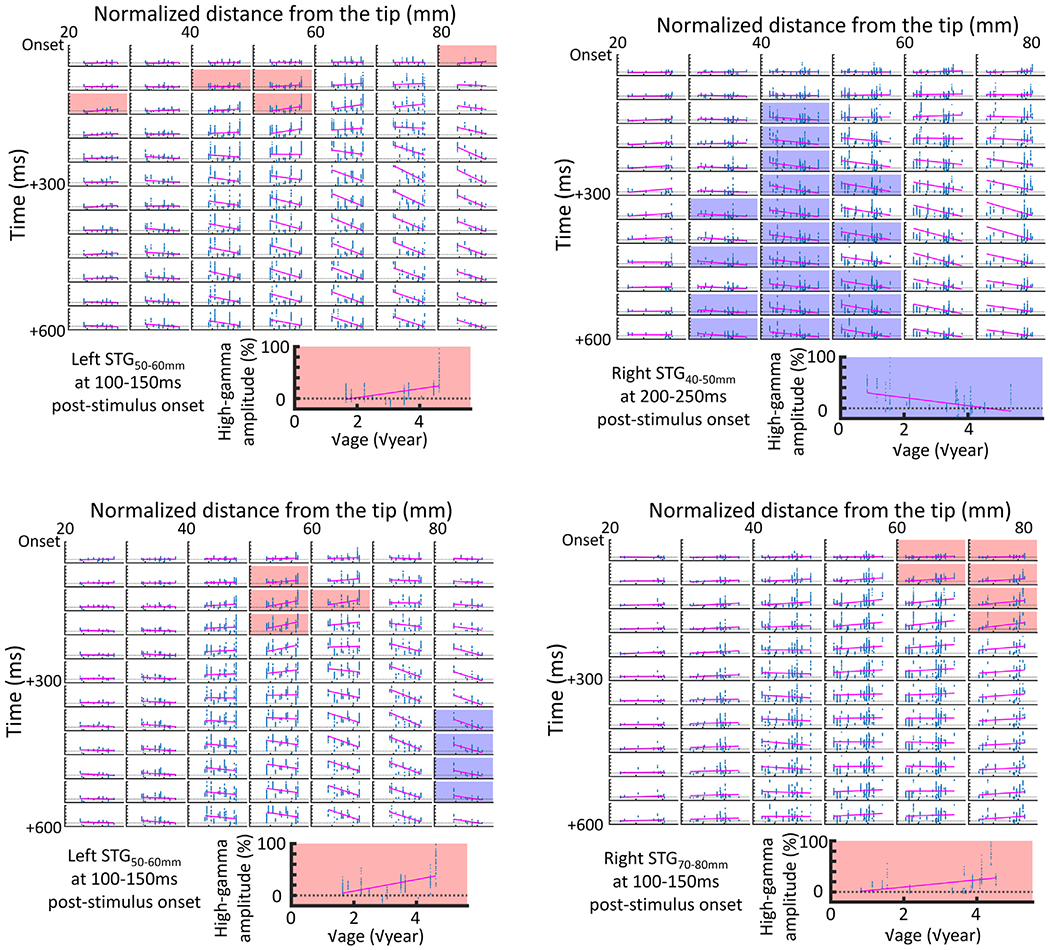
Developmental changes of noise-related high-gamma amplitude responses in the superior temporal gyrus (STG). Each scatter plot shows the relationship between the square-root (√) of age and high-gamma amplitude response at a given region of interest (ROI) in the STG. X-axis: √age of a given patient (√year). Y-axis: High-gamma amplitude response (% change). Pink line: Univariate linear regression line in the model with √age treated as the independent variable and high-gamma amplitude response treated as the dependent variable. Scatter plots highlighted by red- and blue-colored backgrounds denote the timing and ROI showing significant positive and negative effects of √age on the degree of high-gamma augmentation, respectively, with the independent effects of sleep state, clinical profiles, and epilepsy-related variables controlled by the mixed model analysis (Fig. 7). Zoomed is one of the plots showing a significant correlation between √age and high-gamma amplitude responses on both univariate linear regression and mixed model analyses. (Upper) Noise-related high-gamma responses in the left and right STG. (Lower) Speech sound-related high-gamma responses in the left and right STG. Note that the cluster-based test was employed to correct for 84-time comparisons for the left STG analysis and 72 times for the right STG.

Univariate regression analysis clarified the relationship between the square-root of age ( √age: independent variable) and sound-related high-gamma responses at a given analysis mesh (dependent variable) within a given STG ROI of a given hemisphere (Fig. 7–8). We incorporated ‘√age’ in the regression model because investigators have suggested that structural and hemodynamic changes supporting perceptual and cognitive development take place drastically during early childhood and mildly during late childhood, and √age was expected to track the nonlinear processes better than age (Dosenbach et al., 2010; Petanjek et al., 2011; Ullman et al., 2014; Piochon et al., 2016). For interested readers, we employed an ancillary analysis and provided the scatter plots showing the relationship between patient age (instead of √age) and high-gamma dynamics at given STG ROIs (Fig. S2–S6).

Mixed model analysis (Sonoda et al., 2021) subsequently determined ‘at what ROIs’ and ‘at what time windows’ the developmental changes (i.e., enhancement or diminution) in sound-related high-gamma responses remained significant with the independent effects of sleep state, clinical profiles, and epilepsy-related variables controlled. We employed the MATLAB fitlme command (https://www.mathworks.com/help/stats/fitlme.html) to fit the mixed models specified by the following formula: ’high-gamma responses ~ 1 + age + sex + seizure onset zone + MRI + sleep + number of antiseizure medications + (1∣patient)’. We employed mixed model analysis for all ROIs at each 50-ms time window in which iEEG data were collected at least from seven patients and the permutation test revealed significant high-gamma augmentation. The dependent variable was the high-gamma responses at a given analysis mesh (% change in amplitude compared to the baseline). The fixed effect predictors included [1] √age at surgery ( √year), [2] sex (female = 1), [3] presence of seizure onset zone in the temporal lobe (yes = 1), [4] presence of MRI-visible structural lesion (yes = 1), [5] sleep state during sound presentation (sleep = 1; wakefulness = 0), and [6] number of oral antiseizure medications taken immediately before the intracranial electrode implantation. We considered a larger number of antiseizure medications to reflect a more severe seizure-related cognitive burden. The need for polytherapy is generally associated with an increased risk of more disabling seizures and seizure-related cognitive dysfunction (Kwan and Brodie, 2001; Kuroda et al., 2021). To our best knowledge, no single neuropsychological assessment can be employed to quantify the cognitive impairment of individuals ranging from infants to adults. The random effect factors included the intercept and patient. We considered a potential non-independence of high-gamma responses within a given patient and a random high-gamma difference between a patient and others.

We defined a significant developmental change in high-gamma responses as those satisfying the following criteria: [i] the univariate regression analysis showed a correlation between √age and high-gamma amplitude on the cluster-based permutation test (*n* = 1000; cluster size threshold *α* = 0.05) and [ii] the mixed √age effect survived the cluster-based permutation test (*n* = 1000; cluster size threshold *α* = 0.05). We believed that the aforementioned criteria would reduce the risk of Type I error in identifying meaningful √age-dependent high-gamma patterns. We performed the cluster-based test to correct for multiple comparisons across space and time (i.e., number of STG ROIs showing significant high-gamma augmentation × number of 50-ms time windows; Fig. 7; Hagler et al., 2006; Leritz et al., 2011; Han et al., 2013; Baumgarten et al., 2021). To this end, we identified a cluster including ∣ √age effect t-value ∣ >1.96 in a given time-location matrix. We then computed a ‘summation of ∣ √age effect t-value ∣ within a given cluster’ (t_sum_). We subsequently created 1000 permutation data sets with the timing and location of high-gamma amplitude randomly shuffled and computed a ‘summation of ∣ √age effect t-value ∣ within the largest cluster’ (t_permutation-sum_ ) for each permutation data set. Thereby, a cluster of √age effect associated with t_sum_ greater than 95% of the distribution of 1000 t_permutation-sum_ was considered significant. Analysis of high-gamma responses to signal-correlated noises addressed [**Aim 1**] ; specifically, we determined whether noise-related STG neural cost at an early post-stimulus period would be enhanced, in an age-dependent manner but diminished at a delayed period. Analysis of high-gamma responses to speech sound stimuli addressed [**Aim 2**]; specifically, we determined whether older individuals would show diminished speech sound-related posterior STG high-gamma augmentation during a delayed period. For interested readers, we performed ancillary analysis and visualized the effect of √age on high-gamma responses to forward (Fig. S7–S9) and backward-played speech sounds (Fig. S7, S10, S11). We used Matlab R2020 (MathWorks Inc., Natick, MA) for all statistical analyses.

### 2.7. Data availability

All iEEG data and the MATLAB-based codes are available upon request to the corresponding author.

## 3. Results

### 3.1. Behavioral observations

A total of 32 patients (aged 8 months to 28 years) satisfied the eligibility criteria (Table 1). None of the patients had adverse events during the sound presentation. Twenty-two patients were awake, whereas the remaining ten were asleep, and the independent effect of sleep state on high-gamma responses to sound stimuli was controlled for in the mixed model analysis above.

### 3.2. Visualization of sound-related high-gamma responses

Video S1 and Fig. 5 contrast the spatiotemporal dynamics of group-level, sound-related high-gamma amplitude responses between signal-correlated noises and speech sound stimuli. The STG in both hemispheres responded to auditory stimuli with high-gamma augmentation that was initially comparable across both types, but speech sound-related augmentation was sustained longer than signal-correlated noises.

**[Aim 1]** The ROI-based analysis revealed that STG high-gamma augmentation elicited by signal-correlated noises was significantly smaller than that elicited by speech sounds at a delayed period. Initially, the permutation test revealed that high-gamma activity was significantly augmented by both speech sounds and noises in the bilateral STG within 90 ms post-stimulus onset; however, at ≥360 ms post-stimulus onset, speech sounds sustained greater high-gamma responses, compared to signal-correlated noises (Fig. 6 and S1). The maximum amplitude difference (% change) between signal-correlated noises and speech sound stimuli was 26.7% in the left STG (95% confidence interval [CI]: 14.7% to 38.6%) and 21.7% in the right STG (95%CI: 10.5% to 33.0%).

### 3.3. Developmental changes in noise-related high-gamma responses

[**Aim 1**] Fig. 7 shows the mixed model effect of √age (percent change/ √year) on high-gamma amplitude at given 50-ms time windows and STG ROIs. Noise-related high-gamma augmentation in the left STG_40–60 mm_ at an early period was significantly enhanced as a function of √age (maximum mixed model effect: +16.6%/ √year; 95%CI: +5.1 to +28.2%/ √year; t-value: +2.8; Fig. 7A; Table S1). In turn, noise-related high-gamma augmentation in the right STG_30–60 mm_ at a delayed period was significantly diminished (minimum mixed model effect: −12.6%/ √year; 95%CI: −17.0 to −8.1%/ √year; t-value: −5.6; Fig. 7B; Table S2). Video S2 presents the impact of √age on noise-related high-gamma responses. We have provided the scatter plots clarifying the relationship between patient age and noise-related high-gamma responses at left (Fig. 8A) and right STG ROIs (Fig. 8B).

Based on the results of ancillary mixed model analysis incorporating patient ‘age’ (instead of ‘ √age’), we presented the age-dependent trajectories of noise-related high-gamma responses at given STG ROIs ( Fig. S2–S4). Specifically, early augmentation in the left STG_40–60 mm_ was enhanced, as a function of patient age (maximum mixed model effect: +3.7%/year; 95%CI: + 0.04 to + 7.4%/year; t-value: +2.0); thereby, we found that ‘ √age’ and ‘age’ were equally fitted to the linear regression line (mean r^2^ : 0.096 vs 0.095; mean r^2^ difference: 0.001 [95%CI: −0.010 to 0.013]). Alternatively, delayed augmentation in the right STG_30–60 mm_ was diminished as a function of patient age (minimum mixed model effect: −2.0%/year; 95%CI: −2.9 to −1.1%/year; t-value: −4.5); thereby, ‘ √age’ was found to be better fitted to the linear regression line compared to ‘age’ (mean r^2^ : 0.12 vs 0.094; mean r^2^ difference: 0.022 [95%CI: 0.013 to 0.031]).

### 3.4. Anterior-to-posterior functional division within the superior temporal gyrus (STG)

[**Aim 2**] The ROI-based analysis of speech sound-related high-gamma responses demonstrated an anterior-to-posterior STG functional division, as reported in previous iEEG studies (Ozker et al., 2017, 2018; Hamilton et al., 2018). Specifically, the left STG_40–80 mm_ showed *sustained* speech sound-related high-gamma augmentation within 90 ms post-stimulus onset and lasting until sound stimulus offset (maximum augmentation: +53.6% at 60–70 mm and at 400 ms post-stimulus onset; 95%CI: +30.0% to +77.2%). In contrast, the left STG_80–90 mm_ showed transient augmentation lasting until 360 ms post-stimulus onset (maximum augmentation: +19.8% at 220 ms post-stimulus onset; 95%CI: +1.0% to +38.5%; Fig. 6 **and** S1).

Our analysis failed to localize a distinct anterior-to-posterior STG functional division in the right hemisphere. Right STG_40–80 mm_ showed speech sound-related high-gamma augmentation lasting until the stimulus offset (Fig. 6 **and** S1).

### 3.5. Developmental changes in speech sound-related high-gamma responses

[**Aim 2**] The mixed model analysis revealed a double dissociation between the left STG locations and developmental changes in high-gamma responses. The left STG_50–70 mm_ showed √age-dependent enhancement of high-gamma responses at 50–200 ms post-stimulus onset (maximum mixed model effect: + 27.8%/ √year; 95%CI: + 7.4 to +48.3%/ √year; t-value: +2.7; Fig. 7C; Table S3). Conversely, the left STG_80–90 mm_ showed age-dependent diminution of high-gamma responses at 350–550 ms post-stimulus onset (minimum mixed model effect: −58.5%/ √year; 95%CI: −95.7 to −21.3%/ √year; t-value: −3.1; Fig. 7C; Table S4). Video S2 presents the effect of √age on speech sound-related high-gamma responses. We have provided scatter plots clarifying the relationship between patient √age and speech sound-related high-gamma responses at left (Fig. 8C) and right STG ROIs (Fig. 8D).

Based on the results of ancillary mixed model analysis incorporating patient ‘age’ instead of ‘ √age’, we presented the age-dependent trajectories of speech sound-related high-gamma responses at given STG ROIs (Fig. S5–S7). Specifically, early augmentation in the left STG_50–70 mm_ was found to be enhanced as a function of patient age (maximum mixed model effect: +4.4%/year; 95%CI: +1.5 to +7.4%/year; t-value: +3.0); thereby, ‘ √age’ and ‘age’ were equally fitted to the linear regression line (mean r^2^ : 0.16 vs 0.16; mean r^2^ difference: 0.00 [95%CI: −0.001 to 0.01]). Delayed high-gamma augmentation in the left STG_80–90 mm_ was found to be diminished as a function of patient age (minimum mixed model effect: −12.7%/year; 95%CI: −20.7 to −4.7%/year; t-value: −3.1); thereby, ‘ √age’ was better fitted to the linear regression line compared to ‘age’ (mean r^2^: 0.37 vs. 0.31; mean difference: 0.064 [95%CI: 0.038 to 0.091]).

Another ancillary mixed model analysis indicated that increased √age significantly enhanced early forward speech-related high-gamma augmentation in the left STG_50–70 mm_ (maximum mixed model effect: + 36.2%/ √year; 95%CI: +17.7 to +54.7%/ √year; t-value: +3.8; Fig. S8). Increased √age likewise enhanced early backward speech-related high-gamma augmentation, but the degree of √age-dependent enhancement of high-gamma augmentation failed to reach significance (maximum mixed model effect: +19.5%/ √year; 95%CI: −3.0 to +41.9%/ √year; t-value: +1.7; Fig. S10 ) . On the other hand, increased √age diminished delayed forward speech-related high-gamma augmentation in the left STG_80–90 mm_ with significance (minimum mixed model effect on forward speech: −65.0%/ √year; 95%CI: −106.2 to −23.9%/ √year; t-value: −3.1; Fig. S8). Increased √age likewise diminished delayed backward speech-related high-gamma augmentation, but the degree of √age-dependent diminution of delayed backward speech-related high-gamma augmentation failed to survive the cluster-based permutation test (minimum mixed model effect: −52.0%/ √year; 95%CI: −90.9 to −13.1%/ √year; t-value: −2.6; Fig. S10).

## 4. Discussion

### 4.1. Development of early enhancement of and delayed diminution of noise-related stg responses

[**Aim 1**] Our results support the language acquisition view proposed by Bishop (1999; 2000) that through development, the human STG acquires neural dynamics that enable rapid detection and subsequent disregard of acoustic noises. The unique aspect of our iEEG study was the successful clarification of the developmental plasticity of STG high-gamma activations with a temporal window of 50 ms and a spatial resolution of 10 mm. Older individuals had a greater degree of noise-related high-gamma augmentation in the left STG at <200 ms post-stimulus onset (Fig. 7A); both linear and nonlinear regression models equally explained the variance of such early STG high-gamma augmentation (Fig. 8A and S3). Such age-dependent enhancement in early STG high-gamma augmentation may reflect rapid detection of irrelevant acoustic information. In contrast, delayed high-gamma responses in the right STG were found to be more diminished in older individuals (Fig. 7B); this observation may reflect disregard of irrelevant acoustic information. The nonlinear regression model incorporating √age (mean r^2^: 0.12; Fig. 8B) had a better model fitness than that incorporating √age (mean r^2^: 0.094; Fig. S4); this indicates that developmental diminution of delayed STG high-gamma augmentation occurs intensely during infancy/toddlerhood and modestly afterward. The aforementioned developmental trajectories of rapid and delayed noise-related STG high-gamma augmentation can explain the observations that, compared to young children, older children and adults do not expend as much neural cost processing meaningless, irrelevant information such as environmental sounds related to winds, rivers, forests, and traffic (Golestani and Zatorre, 2004; Nittrouer and Lowenstein, 2010; Leech and Saygin, 2011; Klatte et al., 2013). Compared to older children and adults, young children have a greater density of cortical synapses in the STG and greater sensitivity to non-speech auditory stimuli with variable properties (Huttenlocher and Dabholkar, 1997; Albrecht et al., 2000; Paterson et al., 2006). One may attribute developmental reduction of high-gamma responses to noises to the neural pruning in the STG, in which irrelevant cortical synapses are eliminated progressively throughout childhood and adolescence (Huttenlocher and Dabholkar, 1997).

No patients younger than eight months old underwent iEEG recording during the study period. Thus, we were unable to assess the developmental trajectories during early infancy. Both cortical synaptic density and glucose metabolism are suggested to increase during this period (Chugani et al., 1987; Huttenlocher and Dabholkar, 1997). Thus, one may hypothesize that early noise-related high-gamma augmentation would increase as a function of age during early infancy. To the point, the amplitude of initial positive and negative deflections (also known as P1 and N1) evoked by tone stimuli was reportedly greater in toddlers, compared to newborns (Wunderlich et al., 2006).

### 4.2. Development of the anterior-to-posterior functional division in the left stg for speech sound processing

[**Aim 2**] The novel aspect of our iEEG study includes the assessment of developmental trajectories of speech sound-related high-gamma augmentation at each STG ROI. We found that the anterior-to-posterior functional division within the left STG - the basis of the optimal perception of speech sounds - is strengthened after birth. The left STG_40–80 mm_ showed sustained high-gamma augmentation during presentation of speech sounds, which was enhanced in older individuals. This anterior portion of the left STG is suggested to process phonetic information (Hamilton et al., 2018). In older individuals, early detection and processing of speech sounds in the left STG_40–80_ may enable an effective allocation of neural resources to the subsequent auditory perception at a preconscious level (Dehaene et al., 2006). In contrast, the left STG_80–90 mm_ showed transient high-gamma augmentation following speech sound onset, which was diminished intensely during infancy and toddlerhood and modestly afterward. This posterior STG is suggested to detect sentence onsets among a series of words (Hamilton et al., 2018). The developmental trajectory of posterior STG high-gamma augmentation noted in the current study is consistent with the behavioral observation that healthy children begin to use two-word sentences/utterances at around two years of age and subsequently increase the number of words within a sentence in an experience-dependent manner (Howe, 1976).

Based on the overlap of 95%CIs in our ancillary analysis (Fig. S8–S11), we could not make a definitive conclusion that high-gamma responses were differentially sensitive to forward- and backward- speech stimuli. Expressly, the maximum mixed model effect of √age on early forward and backward speech sound-related high-gamma responses in the left STG_50–70 mm_ was +36.2%/ √year (+17.7 to +54.7%/ √year; t-value: +3.8; Fig. S8) and +19.5%/ √year (−3.0 to +41.9%/ √year; t-value: +1.7; Fig. S10), respectively. The effect of √age on delayed high-gamma responses in the left STG_80–90 mm_ was −65.0%/ √year (Fig. S8) and −52.0%/ √year (Fig. S10), respectively. Intelligible semantic content was included in forward but not in backward speech stimuli. Thus, the observed developmental diminution of left STG_80–90 mm_ delayed high-gamma augmentation is difficult to attribute solely to the effect of subconscious semantic processing.

### 4.3. Innovative analysis of the neural dynamics in the developing human brain

We quantified normalized iEEG-based neural responses at STG sites across patients with a wide age range, including children younger than four years. We overcame several fundamental issues related to the inclusion of young children. In general, the temporal lobe poles of young children are incompletely myelinated. Indeed, the surfaces of the temporal lobe poles were erroneously identified in three of the five children aged two to three years included in the present study. We needed to manually delineate the cortical surface for these patients (Fig. 2), and it should be noted that visual inspection remains the gold standard in imaging analysis for defining the cortical surface (Pieters et al., 2013).

Young children have smaller brains than adults (Gerber et al., 2009), so it is plausible to hypothesize that the STG length is likewise shorter in young children, compared to adults. However, we circumvented this issue in the present study, by defining the STG ROIs based on the normalized distance (not absolute distance) from the temporal lobe pole. As a result, regardless of patient age, the left STG_40–50 mm_ was immediately adjacent to the precentral gyrus across the lateral sulcus (Fig. 4A).

### 4.4. Methodological considerations

The present study estimated the developmental trajectories of sound-related neural dynamics based on cross-sectional iEEG analyses because longitudinal iEEG analysis of the same participants over many years is infeasible because of its invasive nature. In addition, high-gamma cortical responses cannot be reliably measured with scalp recording because cortical signals are inevitably obscured by temporal and ocular muscle movement artifacts (Yuval-Greenberg et al., 2008; Carl et al., 2012). Consequently, developmental models can be based on the data from cross-sectional analysis: similar to previous studies that interpreted the developmental changes of cortical synaptic density on postmortem analysis and glucose metabolism on radiotracer positron emission tomography (Chugani et al., 1987; Huttenlocher and Dabholkar, 1997). To minimize the direct, unwanted effect of focal epileptic activity on sound-related high-gamma responses, we excluded the electrode sites affected by the seizure onset zone, interictal spike discharges, or MRI lesions from the analysis. Furthermore, we incorporated multiple potential confounding factors into the mix-model analysis and determined the effects independent of epilepsy-related factors, sex, and sleep state. Antiseizure medications are suggested to suppress cortical excitability and potentially inhibit sensory-related responses (Darmani et al., 2019). The need for polytherapy is generally associated with more severe cognitive delays compared to monotherapy (Kwan and Brodie, 2001). Our mixed model analysis effectively controlled the impact of sleep on sound-related high-gamma responses, whereas previous studies using single-neuron recording and fMRI indicated that auditory perceptual processing is preserved during sleep (Issa et al., 2008; Portas et al., 2000). We reported the developmental effects unattributable to the impact of the epilepsy-related factors, sex, or sleep state incorporated in our mixed model (Fig. 7). Ultimately, one cannot rule out the possibility that the reported effects could be better attributed to an unknown covariate not incorporated in the model.

Our group-level iEEG analysis failed to find cortical sites outside the STG showing significant sound-related high-gamma augmentation. Lack of significance can be attributed to the small sample size at given ROIs. The spatial extent of iEEG sampling is strictly determined by the clinical needs, and we do not place intracranial electrodes for research purposes. None of our study patients had a depth electrode placed within the medial STG, including Heschl’s gyrus, which is suggested to generate sound-related high-gamma augmentation during task-free conditions (Nourski et al., 2015; Hu et al., 2020; Pesnot Lerousseau et al., 2021). iEEG signal sampling using stereotactic depth electrodes can provide a unique window to investigate the maturation of medial-to-lateral functional differentiation of the STG (Bilecen et al., 2002; Leaver et al., 2016; Nourski et al., 2019; Hamilton et al., 2021). Further studies with larger sample sizes are warranted to determine the developmental trajectories of effective connectivity from the medial and lateral STG to other regions (Flinker et al., 2015; Sonoda et al., 2021) and understand how humans learn to transform perceptual information into cognitive and motor representations through development.

## Supplementary Material

1

2

3

4

5

6

7

8

## Figures and Tables

**Table 1:** Patient profile.

Number of patients	32
Mean age in years (range)	11.1 (0.7–28.6)
Proportion of male (%)	53.1
Sampled hemisphere (%)	
Left	37.5
Right	62.5
Seizure onset zone (%)	
involving the temporal lobe	40.6
not involving the temporal lobe	59.4
MRI-visible structural lesion (%)	46.9
Mean number of antiseizure medications (SD)	1.94 (0.619)
Sleep state during sound presentation (%)	
Awake	68.75
Asleep	31.25

SD: Standard deviation.

## Data Availability

All iEEG data and the MATLAB-based codes used in the analyses are available upon request to the corresponding author.

## Abbreviations:

CI: confidence interval
EEG: electroencephalography
ERP: event-related potential
FDR: false discovery rate
fMRI: functional magnetic resonance imaging
iEEG: intracranial electroencephalography
ROI: regions of interest
STG: superior-temporal gyrus
