# Supplementary material for: Developmental organization of neural dynamics supporting auditory perception
Source: Neuroimage. Author manuscript; Available in PMC 2022 Sep 1. (PMC9354710; doi:10.1016/j.neuroimage.2022.119342)
Supplement: 2 [file NIHMS1825576-supplement-2.docx]

**Supplementary document**

in

**Developmental organization of neural dynamics supporting auditory perception**

This document includes

**Fig. S1-S12**

**Tables S1-S4**

**Legends for Videos S1-S3**

**
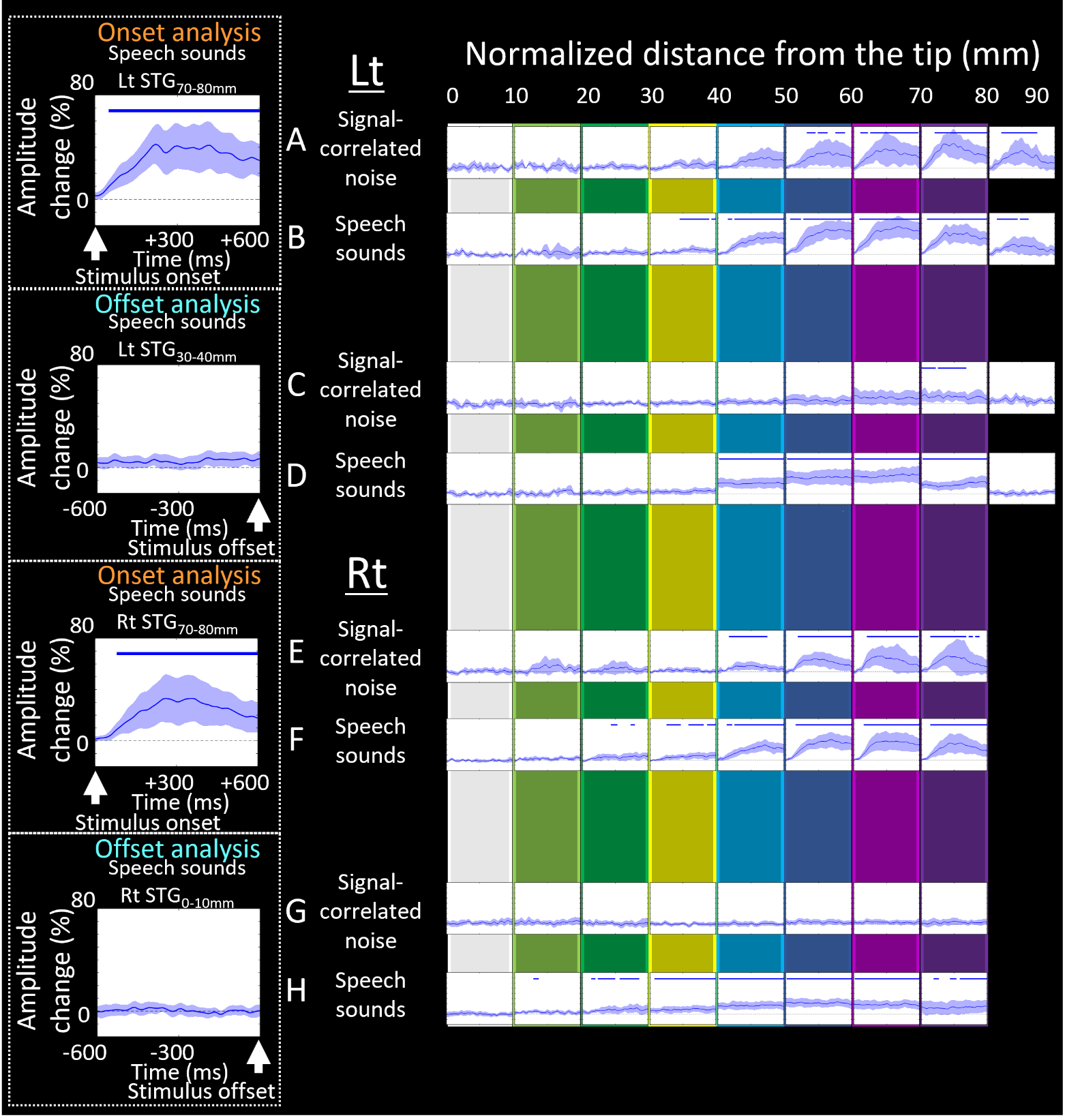
**

**Fig. S1. Sound-related high-gamma modulations in the superior temporal gyrus (STG).**

Noise-related high-gamma amplitude (% change) as a function of time (ms) in the left STG (A and C) and right STG (E and G). Speech sound-related high-gamma amplitude in the left STG (B and D) and right STG (F and H). Upper horizontal bars: Significant amplitude augmentation. The permutation test revealed that high-gamma activity was significantly augmented by speech sound and noises in the bilateral STG within 90 ms post-stimulus onset (A, B, E, F).

**
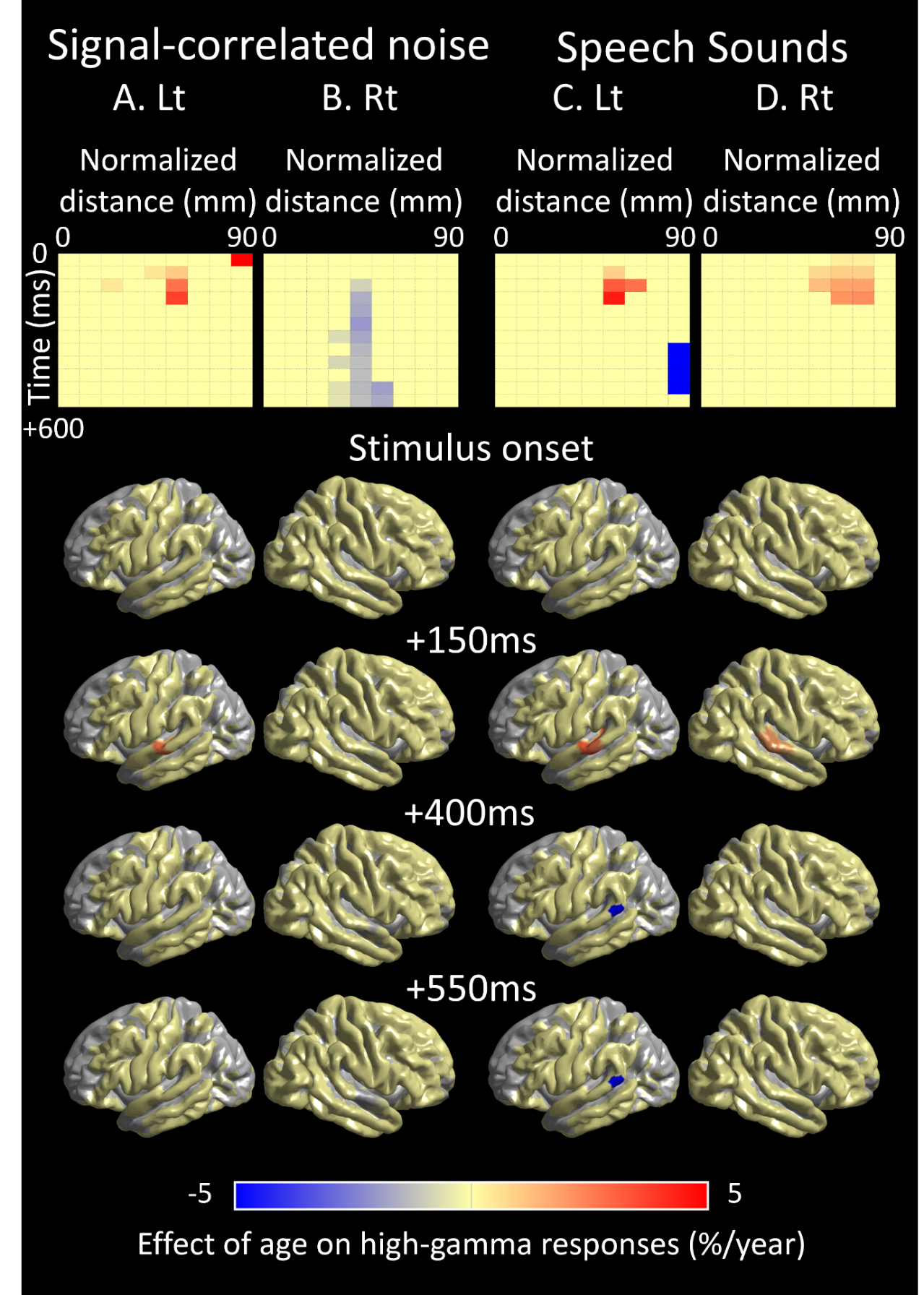
**

**Fig. S2. Developmental changes of sound-related high-gamma dynamics in the superior temporal gyrus (STG).**

Each matrix and brain surface image show the mixed model effect of age (% / year) on high-gamma amplitude at a given 50-ms time window at each STG region of interest (ROI). (A and B) The significant age effect on noise-related high-gamma responses in the left and right STG (see the data source in **Fig. S3 and S4**). (C and D) The significant age effect on speech sound-related high-gamma responses in the left and right STG (see the data source in **Fig. S5 and S6**).

**
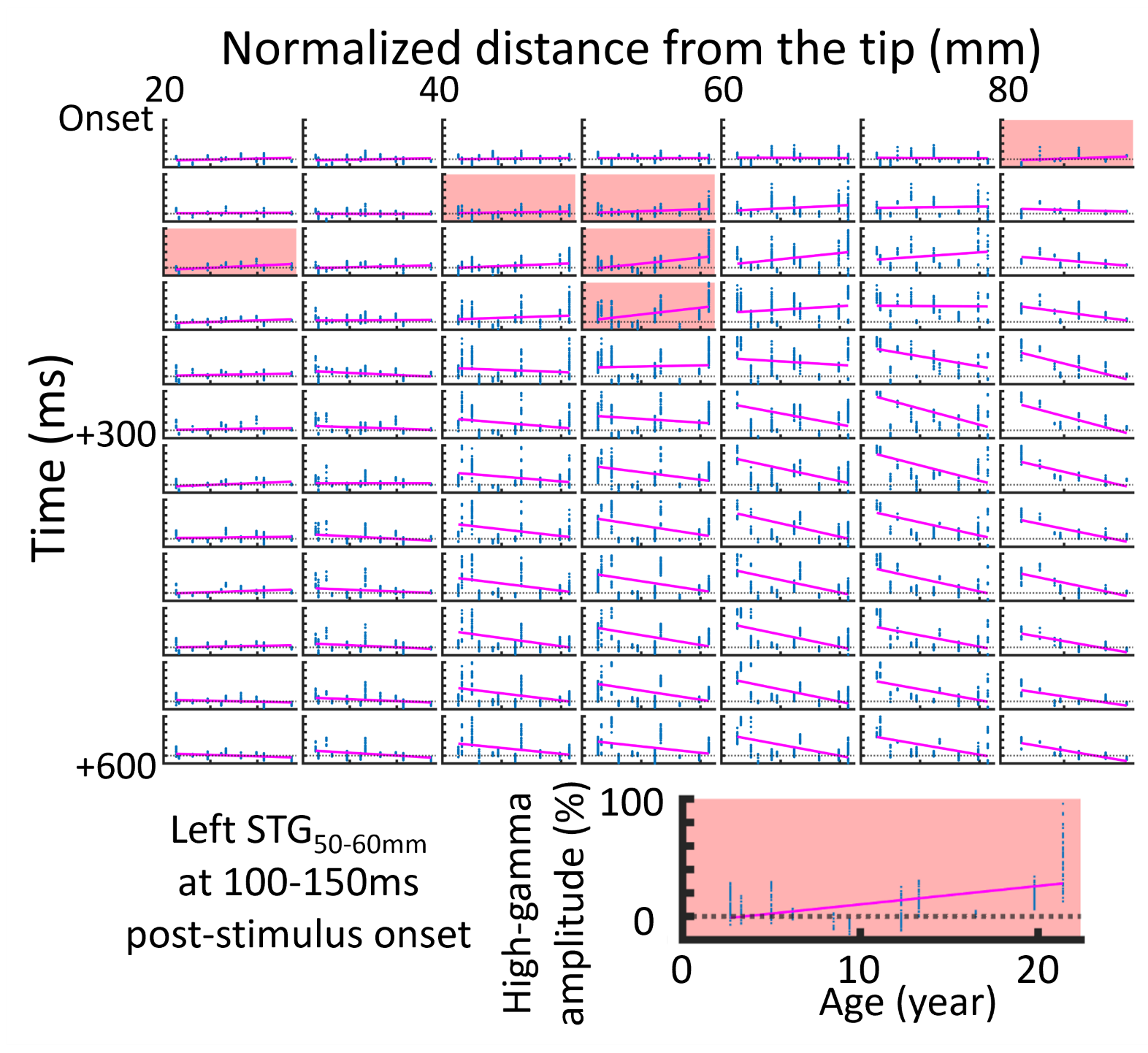
**

**Fig. S3. Developmental changes of noise-related high-gamma amplitude responses in the left superior temporal gyrus.**

Each scatter plot shows the relationship between age and noise-related high-gamma amplitude responses at a given region of interest (ROI) in the left superior temporal gyrus (STG). X-axis: age of a given patient (year). Y-axis: High-gamma amplitude (% change). Pink line: Linear regression line. Scatter plots highlighted by red- and blue-colored backgrounds denote the timing and ROI showing significant positive and negative effects of age on the degree of high-gamma augmentation, respectively, with the independent effects of sleep state, clinical profiles, and epilepsy-related variables controlled by the mixed model analysis (**Fig. S2A**). The zoomed image shows the scatter plot at Left STG_50-60 mm_ (defined as the left STG 50-60 mm normalized distance from the tip) at 100-150 ms post-stimulus onset. The scatter plot in **Fig. 8A** shows the relationship between patient √age (not age) and noise-related high-gamma amplitude responses.

**
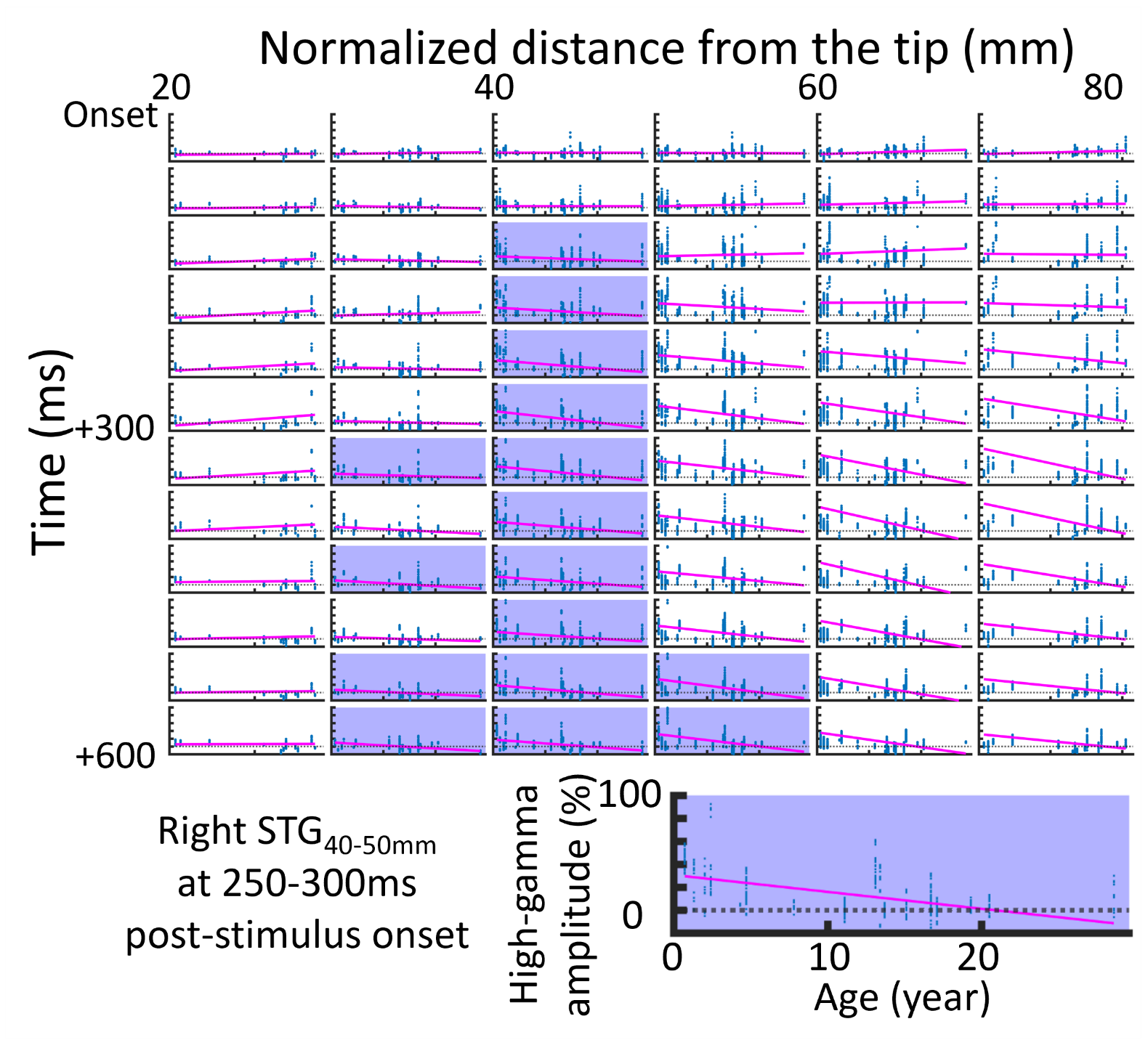
**

**Fig. S4. Developmental changes of noise-related high-gamma amplitude responses in the right superior temporal gyrus.**

Each scatter plot shows the relationship between age and noise-related high-gamma amplitude responses at a given region of interest (ROI) in the right superior temporal gyrus (STG). X-axis: age of a given patient (year). Y-axis: High-gamma amplitude (% change). Pink line: Linear regression line. Scatter plots highlighted by red- and blue-colored backgrounds denote the timing and ROI showing significant positive and negative effects of age on the degree of high-gamma augmentation, respectively, with the independent effects of sleep state, clinical profiles, and epilepsy-related variables controlled by the mixed model analysis (**Fig. S2B**). The zoomed image shows the scatter plot at Right STG_40-50 mm_ (defined as the right STG 40-50 mm normalized distance from the tip) at 250-300 ms post-stimulus onset. The scatter plot in **Fig. 8B** shows the relationship between patient √age (not age) and noise-related high-gamma amplitude responses.

**
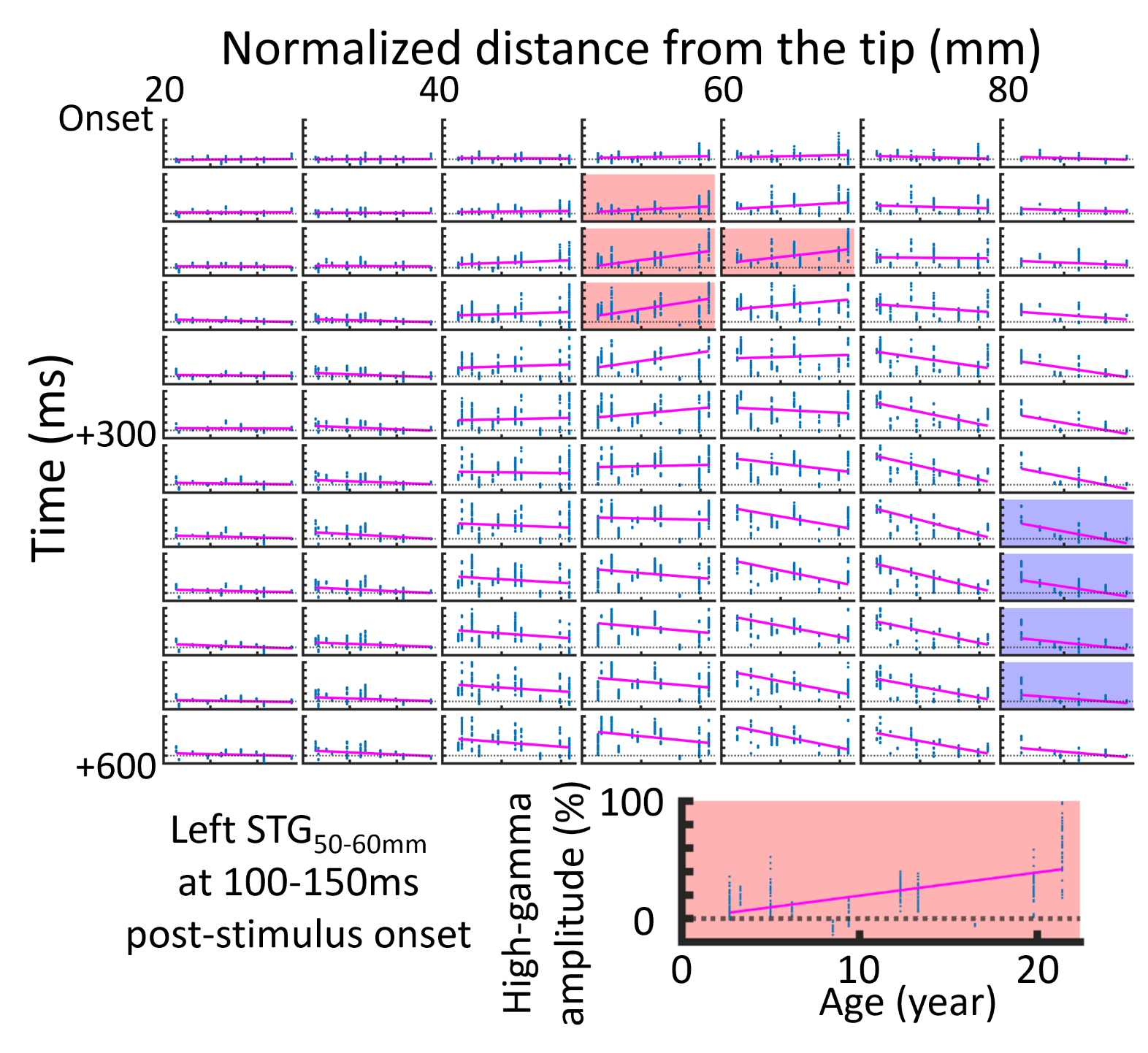
**

**Fig. S5. Developmental changes of speech sound-related high-gamma amplitude responses in the left superior temporal gyrus.**

Each scatter plot shows the relationship between age and speech sound-related high-gamma amplitude responses at a given region of interest (ROI) in the left superior temporal gyrus (STG). X-axis: age of a given patient (year). Y-axis: High-gamma amplitude (% change). Pink line: Linear regression line. Scatter plots highlighted by red- and blue-colored backgrounds denote the timing and ROI showing significant positive and negative effects of age on the degree of high-gamma augmentation, respectively, with the independent effects of sleep state, clinical profiles, and epilepsy-related variables controlled by the mixed model analysis (**Fig. S2C**). The zoomed image shows the scatter plot at Left STG_50-60 mm_ (defined as the left STG 50-60 mm normalized distance from the tip) at 100-150 ms post-stimulus onset. The scatter plot in **Fig. 8C** shows the relationship between patient √age (not age) and speech sound-related high-gamma amplitude responses.

**
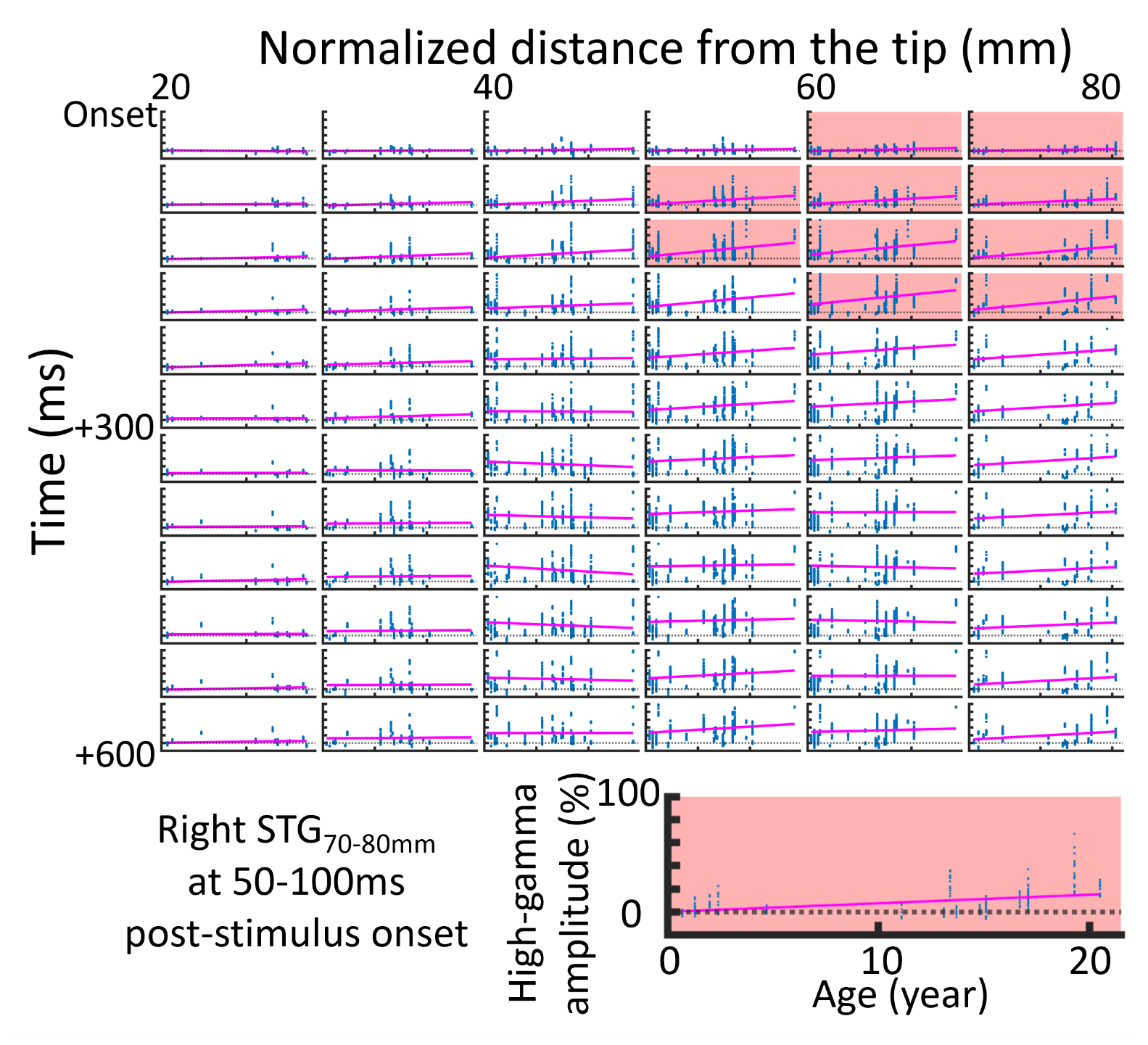
**

**Fig. S6. Developmental changes of speech sound-related high-gamma amplitude responses in the right superior temporal gyrus.**

Each scatter plot shows the relationship between age and speech sound-related high-gamma amplitude responses at a given region of interest (ROI) in the right superior temporal gyrus (STG). X-axis: age of a given patient (year). Y-axis: High-gamma amplitude (% change). Pink line: Linear regression line. Scatter plots highlighted by red- and blue-colored backgrounds denote the timing and ROI showing significant positive and negative effects of age on the degree of high-gamma augmentation, respectively, with the independent effects of sleep state, clinical profiles, and epilepsy-related variables controlled by the mixed model analysis (**Fig. S2D**). The zoomed image shows the scatter plot at Right STG_70-80 mm_ (defined as the right STG 70-80 mm normalized distance from the tip) at 50-100 ms post-stimulus onset. The scatter plot in **Fig. 8D** shows the relationship between patient √age (not age) and speech sound-related high-gamma amplitude responses.

**
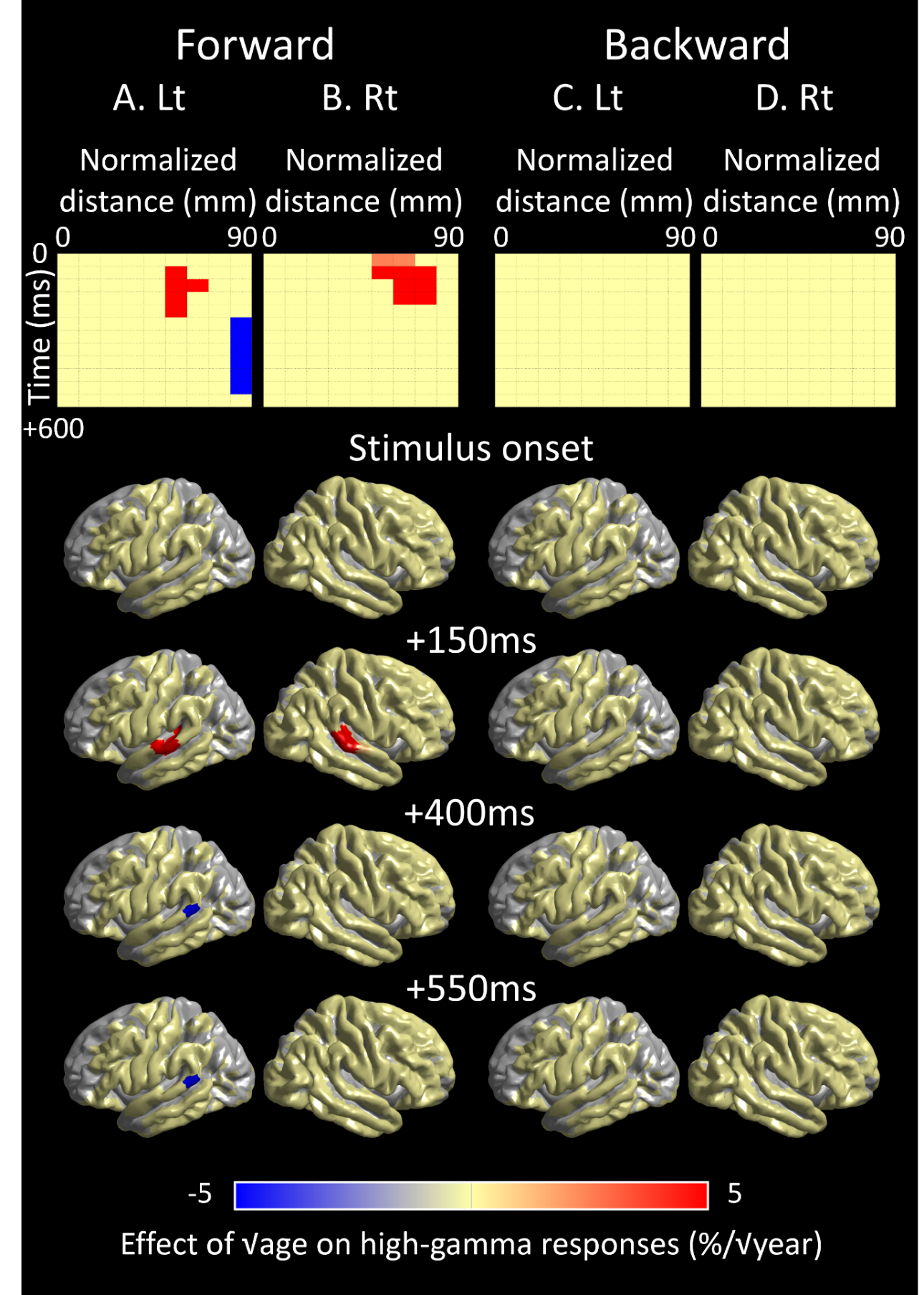
**

**Fig. S7. Developmental changes of sound-related high-gamma dynamics in the superior temporal gyrus (STG).**

Each matrix and brain surface image show the mixed model effect of √age (% / √year) on high-gamma amplitude at a given 50-ms time window at each STG region of interest (ROI). (A and B) The √age effect on forward speech-related high-gamma responses in the left and right STG (see the data source in **Fig. S8 and S9**). (C and D) The √age effect on backward speech-related high-gamma responses in the left and right STG (see the data source in **Fig. S10 and S11**).

**
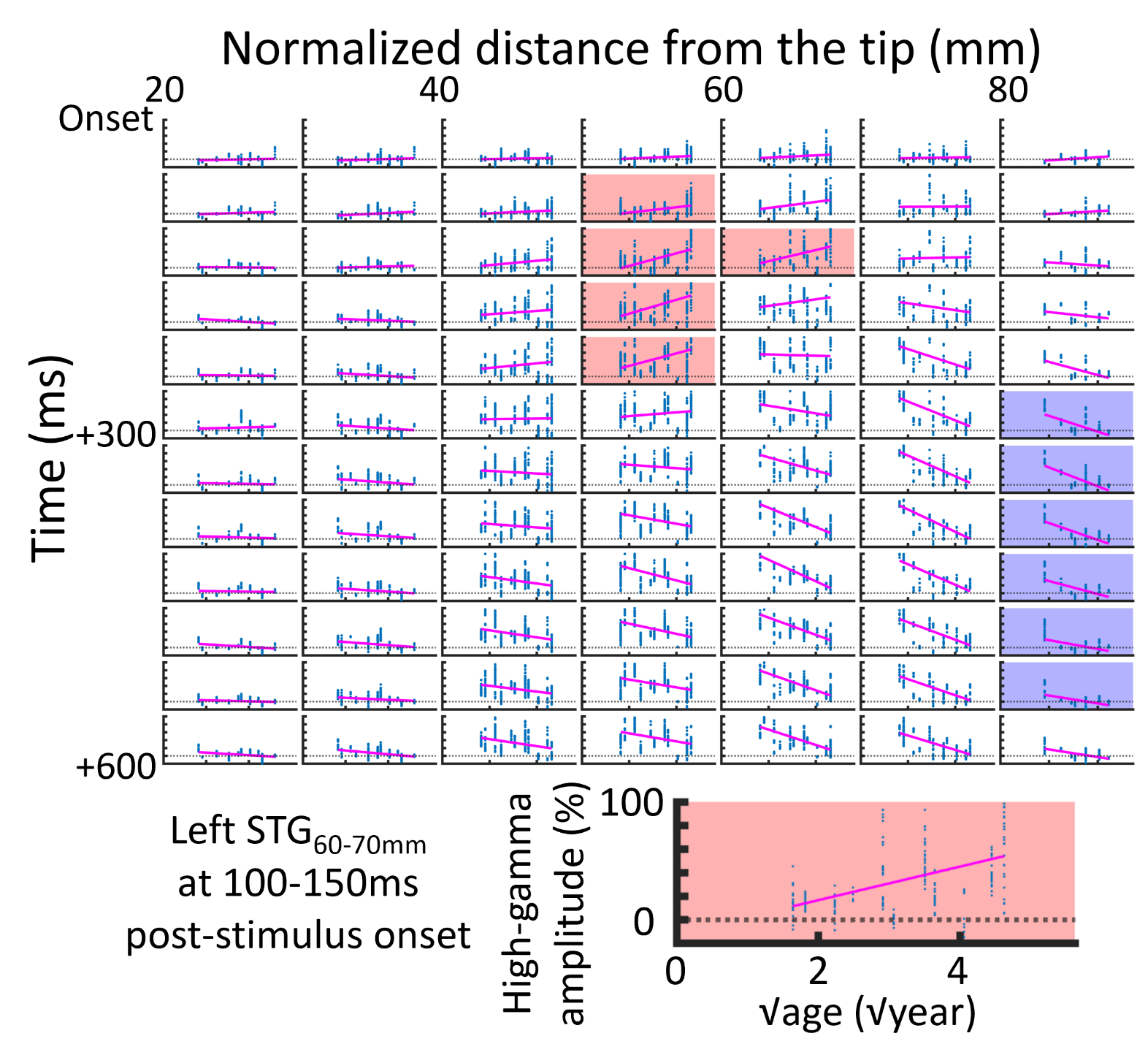
**

**Fig. S8. Developmental changes of forward speech-related high-gamma amplitude responses in the left superior temporal gyrus.**

Each scatter plot shows the relationship between √age and forward speech-related high-gamma amplitude responses at a given region of interest (ROI) in the left superior temporal gyrus (STG). X-axis: √age of a given patient (√year). Y-axis: High-gamma amplitude (% change). Pink line: Linear regression line. Scatter plots highlighted by red- and blue-colored backgrounds denote the timing and ROI showing significant positive and negative effects of √age on the degree of high-gamma augmentation, respectively, with the independent effects of sleep state, clinical profiles, and epilepsy-related variables controlled by the mixed model analysis (**Fig. S7A**). The zoomed image shows the scatter plot at Left STG_60-70 mm_ (defined as the left STG 60-70 mm normalized distance from the tip) at 100-150 ms post-stimulus onset.

**
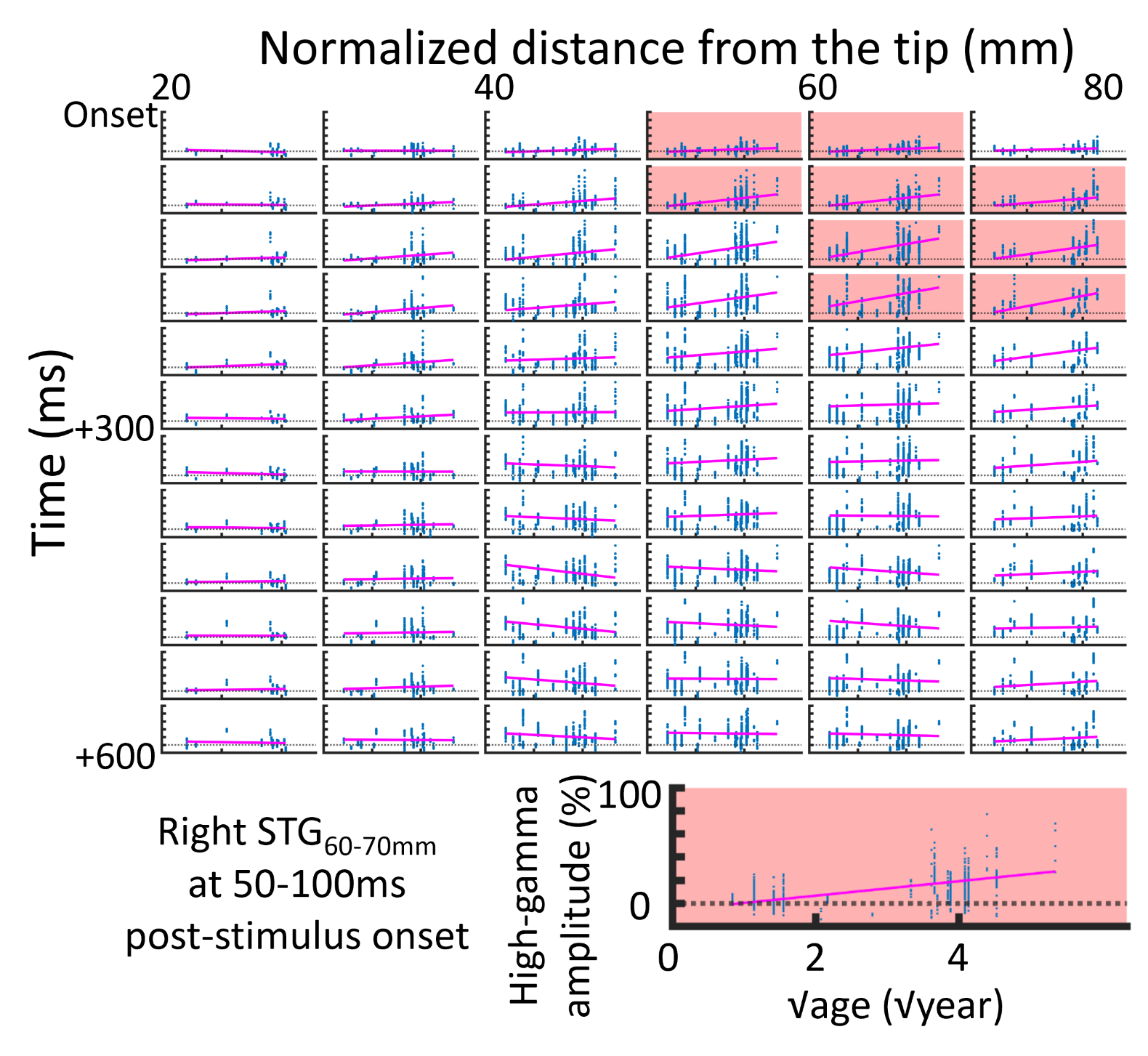
**

**Fig. S9. Developmental changes of forward speech-related high-gamma amplitude responses in the right superior temporal gyrus.**

Each scatter plot shows the relationship between √age and forward speech-related high-gamma amplitude responses at a given region of interest (ROI) in the right superior temporal gyrus (STG). X-axis: √age of a given patient (√year). Y-axis: High-gamma amplitude (% change). Pink line: Linear regression line. Scatter plots highlighted by red- and blue-colored backgrounds denote the timing and ROI showing significant positive and negative effects of √age on the degree of high-gamma augmentation, respectively, with the independent effects of sleep state, clinical profiles, and epilepsy-related variables controlled by the mixed model analysis (**Fig. S7B**). The zoomed image shows the scatter plot at Right STG_60-70 mm_ (defined as the right STG 60-70 mm normalized distance from the tip) at 50-100 ms post-stimulus onset.

**
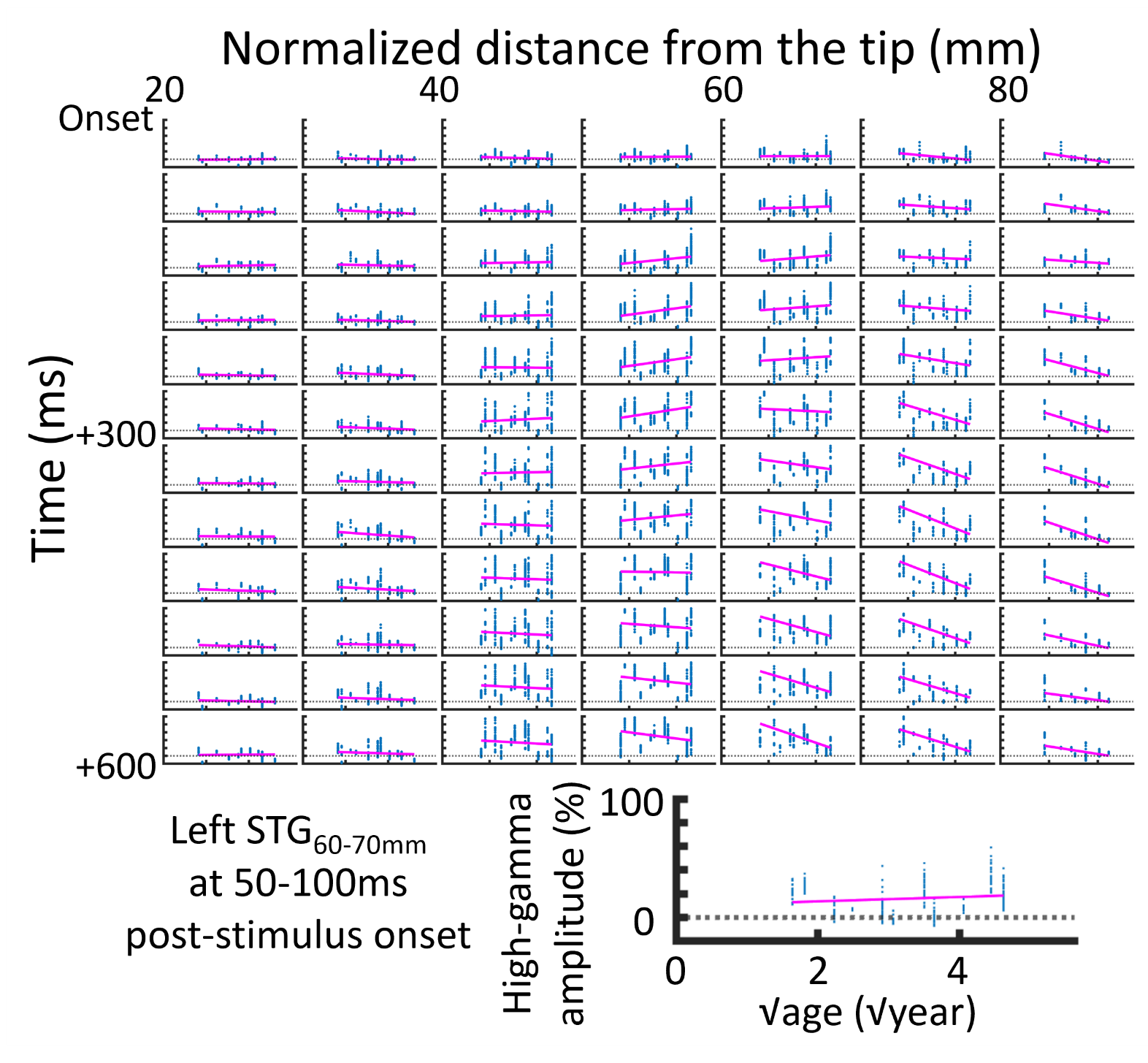
**

**Fig. S10. Developmental changes of backward speech-related high-gamma amplitude responses in the left superior temporal gyrus.**

Each scatter plot shows the relationship between √age and backward speech-related high-gamma amplitude responses at a given region of interest (ROI) in the left superior temporal gyrus (STG). X-axis: √age of a given patient (√year). Y-axis: High-gamma amplitude (% change). Pink line: Linear regression line. The zoomed image shows the scatter plot at Left STG_60-70 mm_ (defined as the left STG 60-70 mm normalized distance from the tip) at 50-100 ms post-stimulus onset.

**
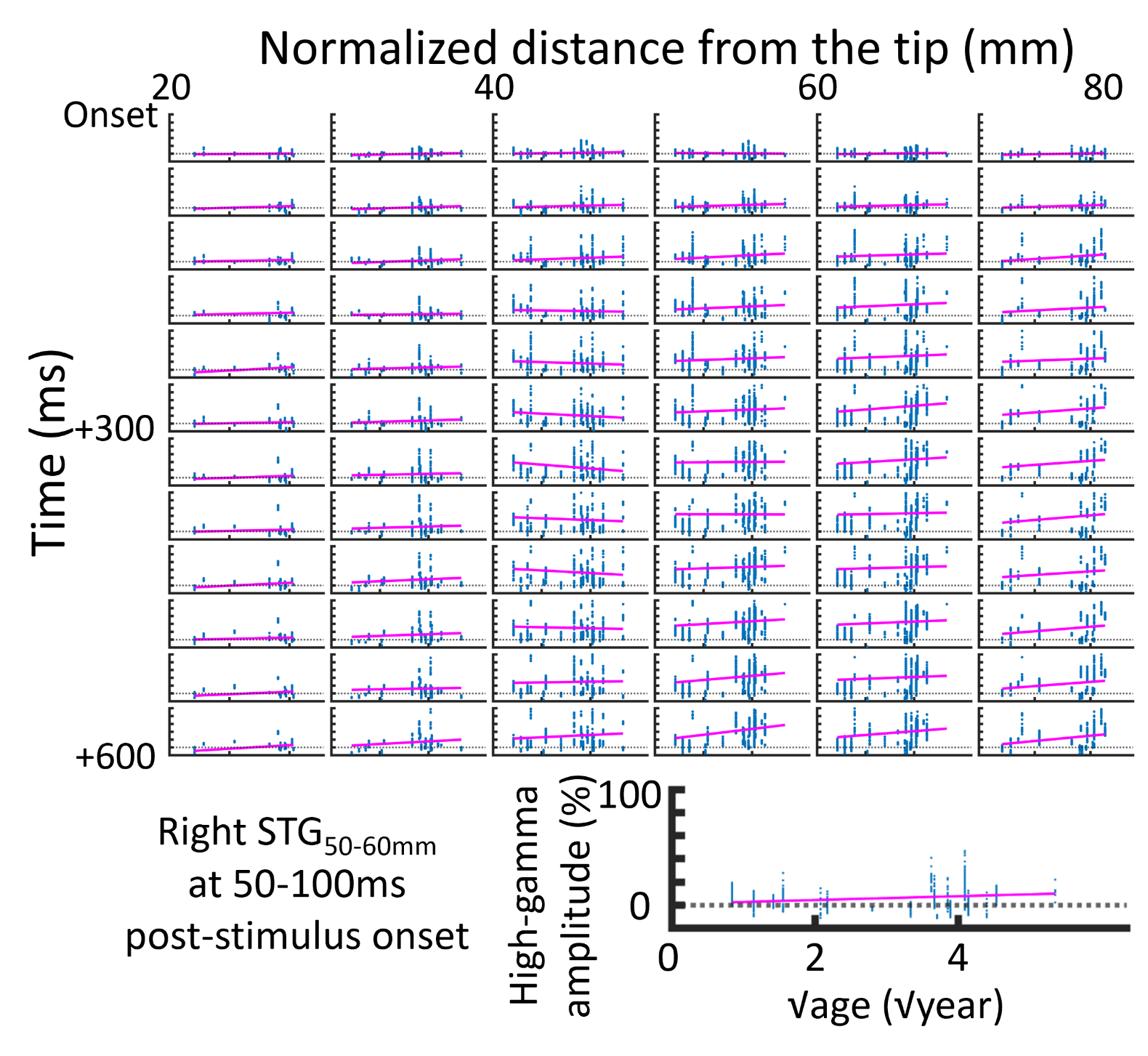
**

**Fig. S11. Developmental changes of backward speech-related high-gamma amplitude responses in the right superior temporal gyrus.**

Each scatter plot shows the relationship between √age and backward speech-related high-gamma amplitude responses at a given region of interest (ROI) in the right superior temporal gyrus (STG). X-axis: √age of a given patient (√year). Y-axis: High-gamma amplitude (% change). Pink line: Linear regression line. The zoomed image shows the scatter plot at Right STG_50-60 mm_ (defined as the right STG 50-60 mm normalized distance from the tip) at 50-100 ms post-stimulus onset.


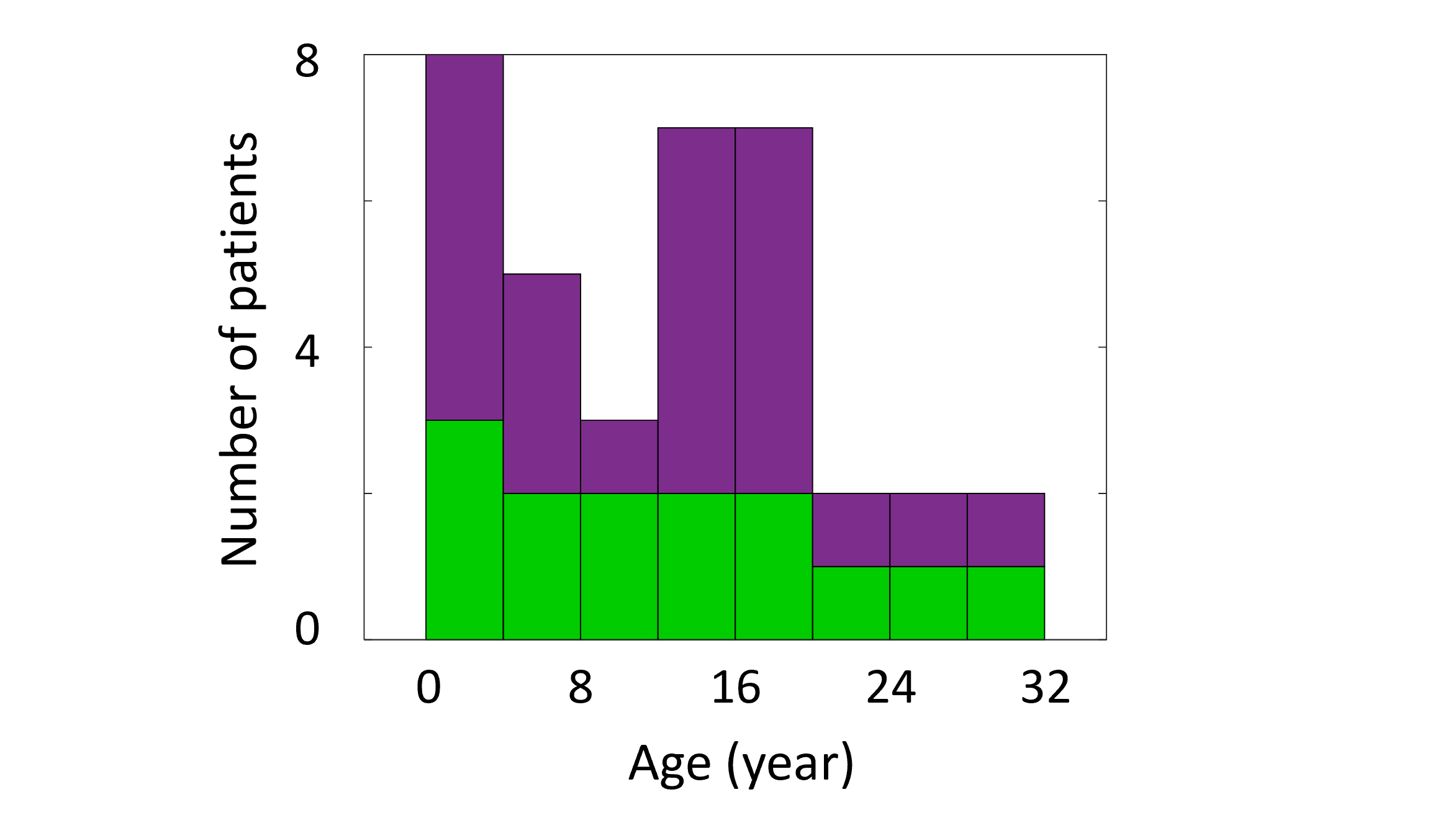


**Fig. S12. Distribution of patient ages.**

Green: left hemisphere. Purple: right hemisphere.

| Variables | Estimate | SE | t-value | Unadjusted p | 95%CI LL | 95%CI UL |
| --- | --- | --- | --- | --- | --- | --- |
| Intercept | -22.9 | 26.9 | -0.9 | 0.40 | -75.6 | 29.9 |
| √age | 16.6 | 5.9 | 2.8 | 0.0048 | 5.1 | 28.2 |
| Sex | -11.4 | 11.9 | -1.0 | 0.34 | -34.8 | 12.1 |
| Seizure onset zone | -5.1 | 10.0 | -0.5 | 0.61 | -24.6 | 14.5 |
| MRI | -29.1 | 13.8 | -2.1 | 0.036 | -56.2 | -1.9 |
| sleep | -26.1 | 21.3 | -1.2 | 0.21 | -67.8 | 15.6 |
| Number of antiseizure medications | 2.0 | 8.6 | 0.2 | 0.82 | -14.9 | 18.9 |

**Table S1. Results of mixed model analysis of noise-related high-gamma responses in the left STG_50-60_ _mm_ at 100-150 ms post-stimulus.** Each one-point increase in √age was associated with enhanced high-gamma augmentation by 16.6%, independently of the covariates. Coefficient of determination for mixed model = 0.76. CI = Confidence interval, LL = Lower limit, SE = Standard error, UL = Upper limit

| Variables | Estimate | SE | t-value | Unadjusted p | 95%CI LL | 95%CI UL |
| --- | --- | --- | --- | --- | --- | --- |
| Intercept | 35.2 | 13.2 | 2.7 | 0.0078 | 9.3 | 61.1 |
| √age | -12.6 | 2.3 | -5.6 | 3.3 x 10^-8^ | -17.0 | -8.1 |
| Sex | -5.4 | 6.0 | -0.9 | 0.37 | -17.2 | 6.4 |
| Seizure onset zone | 9.3 | 5.8 | 1.6 | 0.11 | -2.2 | 20.7 |
| MRI | -12.3 | 5.8 | -2.1 | 0.034 | -23.6 | -0.9 |
| sleep | -7.6 | 5.9 | -1.3 | 0.20 | -19.3 | 4.1 |
| Number of antiseizure medications | 12.5 | 4.7 | 2.6 | 0.0082 | 3.2 | 21.8 |

**Table S2. Results of mixed model analysis of noise-related high-gamma responses in the right STG_40-50 mm_ at 250-300 ms post-stimulus.** Each one-point increase in √age was associated with reduced high-gamma augmentation by 12.6%, independently of the covariates. Coefficient of determination for mixed model = 0.64. CI = Confidence interval, LL = Lower limit, SE = Standard error, UL = Upper limit

| Variables | Estimate | SE | t-value | Unadjusted p | 95%CI LL | 95%CI UL |
| --- | --- | --- | --- | --- | --- | --- |
| Intercept | -16.4 | 47.5 | -0.3 | 0.73 | -109.6 | 76.9 |
| √age | 27.8 | 10.4 | 2.7 | 0.0075 | 7.4 | 48.3 |
| Sex | -33.3 | 21.1 | -1.6 | 0.11 | -74.7 | 8.1 |
| Seizure onset zone | -3.1 | 17.6 | -0.2 | 0.86 | -37.6 | 31.5 |
| MRI | -60.8 | 24.5 | -2.5 | 0.013 | -108.8 | -12.7 |
| sleep | -48.8 | 37.6 | -1.3 | 0.19 | -122.5 | 25.0 |
| Number of antiseizure medications | 4.6 | 15.3 | 0.3 | 0.76 | -25.3 | 34.5 |

**Table S3. Results of mixed model analysis of speech sound-related high-gamma responses in the left STG_50-60 mm_ at 150-200 ms post-stimulus.** Each one-point increase in √age was associated with enhanced high-gamma augmentation by 27.8%, independently of the covariates. Coefficient of determination for mixed model = 0.77. CI = Confidence interval, LL = Lower limit, SE = Standard error, UL = Upper limit

| Variables | Estimate | SE | t-value | Unadjusted p | 95%CI LL | 95%CI UL |
| --- | --- | --- | --- | --- | --- | --- |
| Intercept | 108.8 | 26.5 | 4.1 | 4.8 x 10^-5^ | 56.7 | 160.9 |
| √age | -58.5 | 18.9 | -3.1 | 0.0021 | -95.7 | -21.3 |
| Sex | 63.9 | 25.0 | 2.6 | 0.011 | 14.8 | 113.0 |
| Seizure onset zone | 36.6 | 30.0 | 1.2 | 0.22 | -22.3 | 95.5 |
| MRI | 35.2 | 20.5 | 1.7 | 0.087 | -5.1 | 75.6 |
| sleep | 86.9 | 53.1 | 1.63 | 0.10 | -17.6 | 191.3 |
| Number of antiseizure medications | -0.5 | 9.2 | -0.05 | 0.95 | -18.7 | 17.7 |

**Table S4. Results of mixed model analysis of speech sound-related high-gamma responses in the left STG_80-90_ _mm_ at 350-400 ms post-stimulus.** Each one-point increase in √age was associated with reduced high-gamma augmentation by 58.5%, independently of the covariates. Coefficient of determination for mixed model = 0.89. CI = Confidence interval, LL = Lower limit, SE = Standard error, UL = Upper limit

**Video legend**

**Video S1. Spatiotemporal dynamics of sound-related high-gamma modulations.** Left: Group-level high-gamma modulations elicited by signal-correlated noises. Right: High-gamma modulations elicited by speech sound stimuli (i.e., average during forward and backward speech sound presentations).

**Video S2. Effect of square root of** **age on sound-related high-gamma modulations.** Left: Effect of √age on noise-related high-gamma activity (see the data source in **Fig. 8A and 8B**). Right: Effect of √age on high-gamma activity modulated by speech sound stimuli (i.e., average during forward and backward speech sound presentations; see the data source in **Fig. 8C and 8D**).

**Video S3. Effect of age on sound-related high-gamma modulations.** Left: Effect of age on noise-related high-gamma activity (see the data source in **Fig. S3 and S4**). Right: Effect of age on high-gamma activity modulated by speech sound stimuli (i.e., average during forward and backward speech sound presentations; see the data source in **Fig. S5 and S6**).
