# Supplementary figures and images for: Developmental organization of neural dynamics supporting auditory perception
Source: Neuroimage. Author manuscript; Available in PMC 2022 Sep 1. (PMC9354710; doi:10.1016/j.neuroimage.2022.119342)

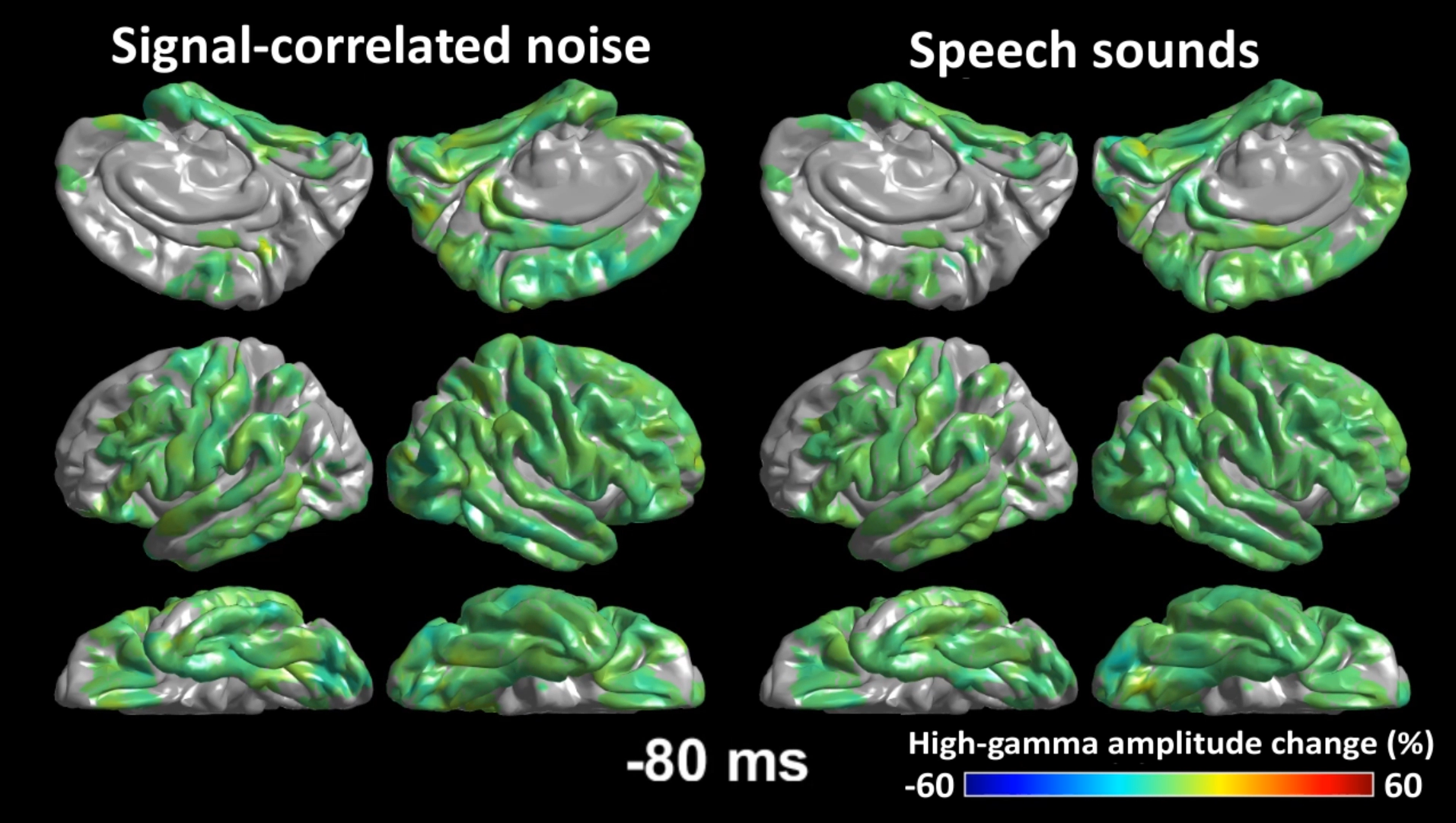

Supplement: 3 [file NIHMS1825576-supplement-3.zip › mmc3.tiff]

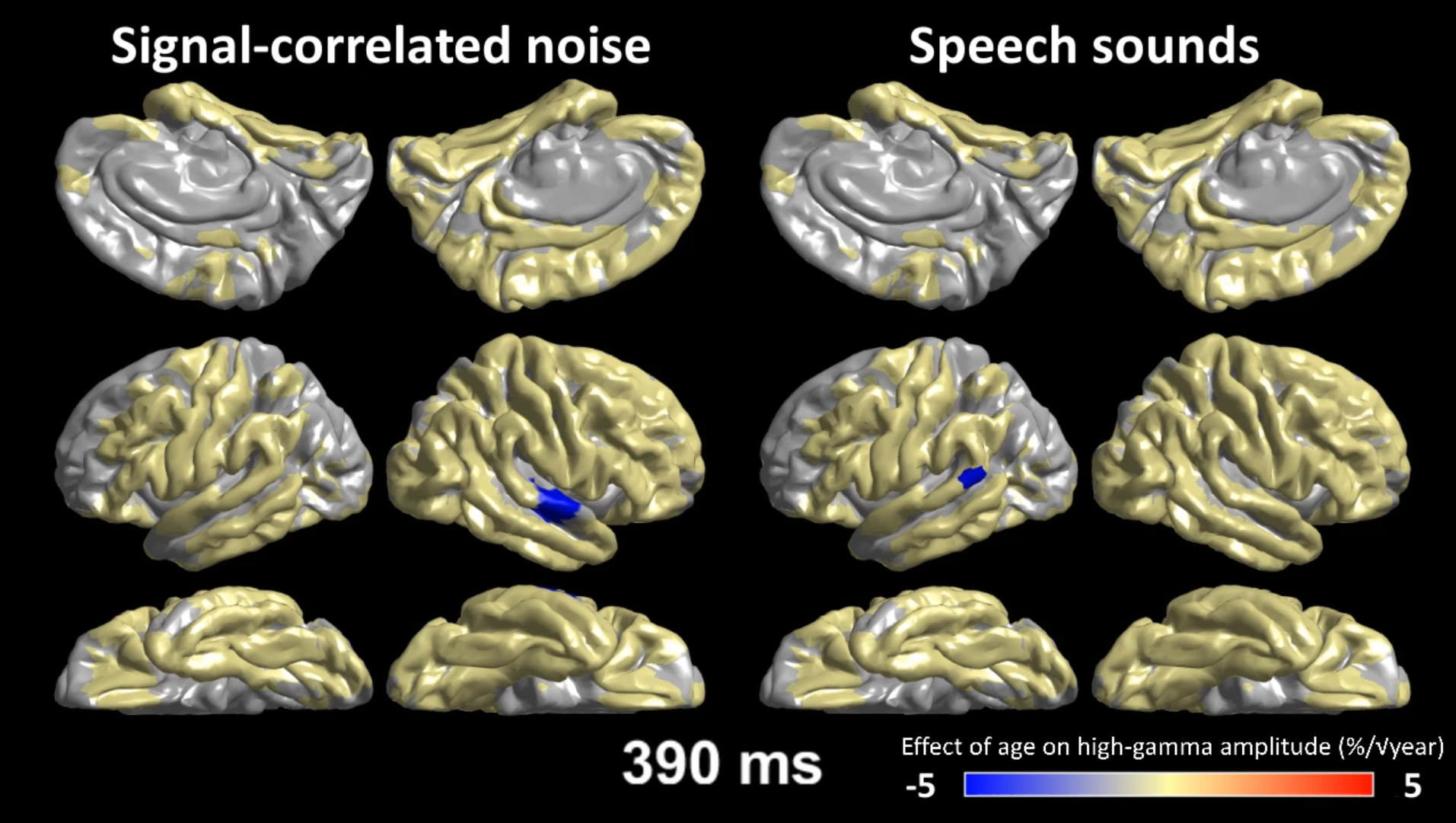

Supplement: 4 [file NIHMS1825576-supplement-4.zip › mmc4.tiff]

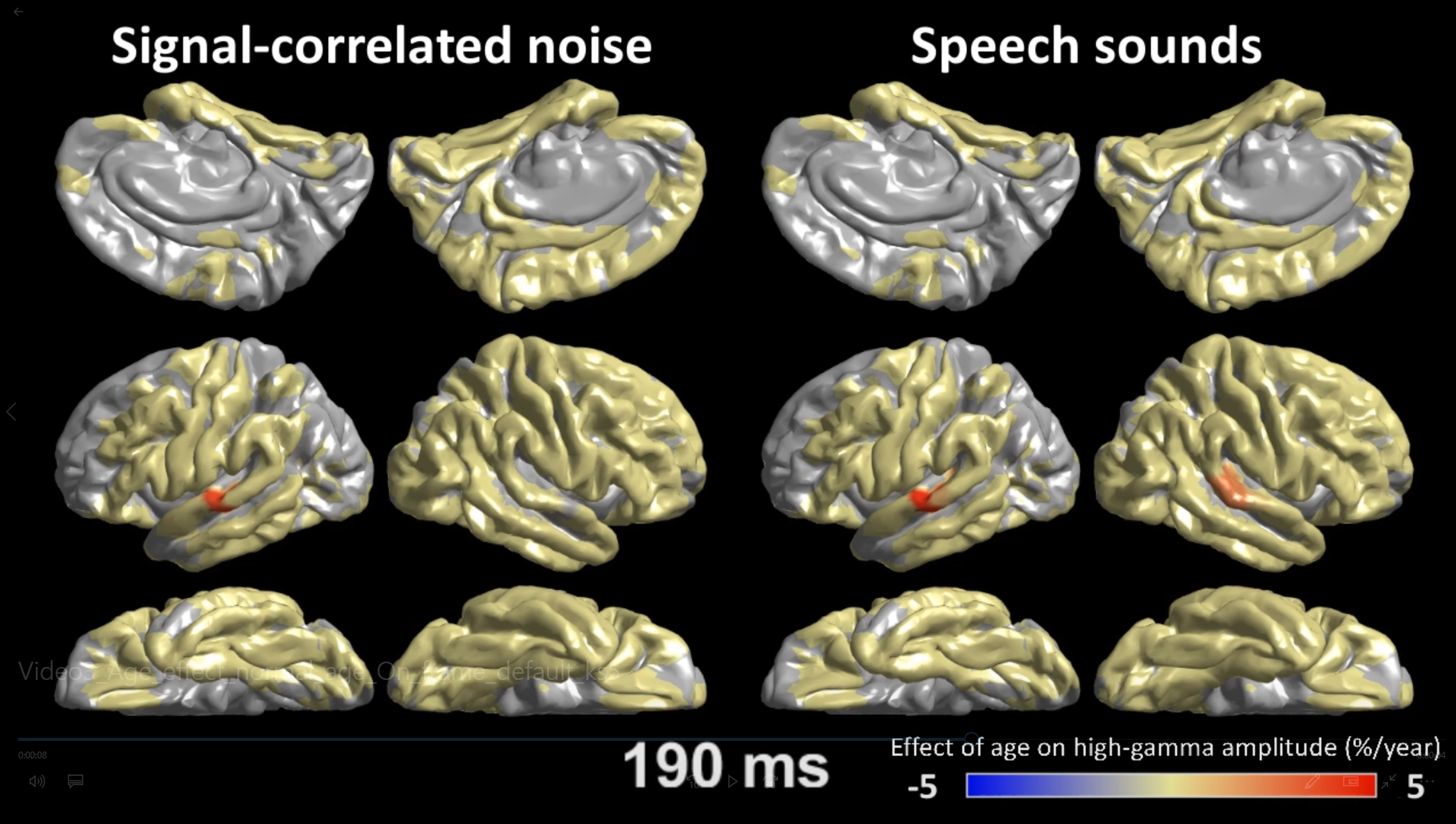

Supplement: 5 [file NIHMS1825576-supplement-5.zip › mmc5.tiff]
